# First Report of Molecular Prevalence and Genetic Characterization of *Enterocytozoon bieneusi* in Young Dairy Cattle in Western Romania

**DOI:** 10.3390/ani16203178

**Published:** 2026-10-10

**Authors:** Mirela Imre, Tamás Szűts, Marius-Stelian Ilie, Sorin Morariu, Gheorghe Dărăbuș, Doru Morar, Tiana Florea, Corina Badea, Alexandra Pocinoc, Alexandru Mladin, Kálmán Imre

**Affiliations:** 1 Faculty of Veterinary Medicine, University of Life Sciences “King Mihai I” from Timisoara, 300645 Timisoara, Romania; mariusilie@usvt.ro (M.-S.I.); sorinmorariu@usvt.ro (S.M.); gheorghedarabus@usvt.ro (G.D.); dorumorar@usvt.ro (D.M.); tijana.florea@usvt.ro (T.F.); corina.badea@usvt.ro (C.B.); alexandra.pocinoc@usvt.ro (A.P.); alexandru.mladin.fmv@usvt.ro (A.M.); kalmanimre@usvt.ro (K.I.); 2 Diversity and Evolution Research Group, Department of Zoology, University of Veterinary Medicine Budapest, Rottenbiller utca 50, 1071 Budapest, Hungary; szuts.tamas@univet.hu

**Keywords:** microsporidia, *E. bieneusi*, cattle, molecular epidemiology, genotyping, zoonosis, Romania

## Abstract

*Enterocytozoon bieneusi* is an important microsporidian pathogen with a broad host range and potential zoonotic relevance, yet molecular data from cattle in Romania are lacking. This study investigated, for the first time, the molecular prevalence, genotype diversity, and phylogenetic relationships of *E. bieneusi* in young dairy cattle in western Romania. A total of 190 fecal samples were collected from young dairy cattle, comprising pre-weaned calves and post-weaned cattle up to 12 months of age from 28 farms in Arad and Timiș counties and examined by nested PCR targeting the internal transcribed spacer (ITS) region of the rRNA gene. *E. bieneusi* DNA was detected in 27/190 cattle, corresponding to an overall prevalence of 14.2% (95% CI: 10.0–19.9%). Pre-weaned cattle showed a higher prevalence than post-weaned animals (18.1% vs. 8.1%), but the difference was not statistically significant (OR = 2.51, 95% CI: 0.96–6.54; *p* = 0.058). High-quality ITS sequences were obtained from 20 positive samples, revealing five established genotypes, namely BEB4 (45.0%), I (25.0%), J (10.0%), BEB8 (10.0%), and BEB17 (10.0%), among the genotyped isolates (*n* = 20). Phylogenetic analysis placed all Romanian isolates within Group 2, predominantly associated with ruminants but also containing genotypes reported in humans. These findings establish the first molecular baseline for *E. bieneusi* in young dairy cattle in western Romania and provide initial information on their epidemiological relevance within a One Health framework.

## Simple Summary

*Enterocytozoon bieneusi* is an important intestinal microsporidian parasite that infects a wide range of animal species and has also been reported in humans. Cattle may contribute to the environmental dissemination of this parasite, and some *E. bieneusi* genotypes identified in cattle have also been reported in humans. However, molecular information on the occurrence and genetic diversity of *E. bieneusi* in cattle in Romania is currently lacking. Therefore, the present study investigated the occurrence of *E. bieneusi* in young dairy cattle from western Romania and characterized the parasite genetically using molecular methods targeting the internal transcribed spacer region of the ribosomal RNA gene. The study provides the first molecular data on *E. bieneusi* in young dairy cattle, comprising pre-weaned calves and post-weaned cattle up to 12 months of age and contributes to the understanding of its genetic diversity and potential epidemiological significance in cattle. The findings may provide a useful baseline for future investigations of *E. bieneusi* in Romanian livestock and support a One Health perspective on the circulation of potentially zoonotic genotypes.

## 1. Introduction

*Enterocytozoon bieneusi* is an obligate intracellular microsporidian fungus-related parasite with a broad host range and is recognized as the most frequently diagnosed microsporidian species infecting humans and animals worldwide [1,2,3]. Although infections are often asymptomatic or self-limiting in immunocompetent individuals, *E. bieneusi* may cause chronic diarrhea, malabsorption and severe disease in immunocompromised patients, making it an important opportunistic pathogen of both medical and veterinary significance [1]. The parasite has been reported in numerous domestic and wild animal species as well as in environmental samples, supporting its importance within the One Health framework [4,5].

Transmission occurs primarily through the fecal–oral route via environmentally resistant spores contaminating water, food and the environment [5]. Consequently, livestock play an important role in the epidemiology of *E. bieneusi*, acting as reservoirs for environmental dissemination and potential zoonotic transmission [6]. Among domestic animals, cattle have attracted particular attention because of their worldwide distribution, intensive production systems and frequent contact with humans, all of which facilitate the circulation of the parasite between animals, the environment and farm workers [4,5].

The internal transcribed spacer (ITS) region of the rRNA gene is the accepted molecular marker for genotyping *E. bieneusi* because of its high sequence polymorphism and discriminatory power [7]. To date, more than 600 ITS genotypes have been identified and classified into 15 phylogenetic groups, reflecting the remarkable genetic diversity of this pathogen [8]. Groups 1 and 2 contain most genotypes with confirmed or potential zoonotic significance, whereas the remaining groups are composed predominantly of host-adapted genotypes [8]. However, Group 2 membership alone does not establish zoonotic risk, as some genotypes are predominantly host-adapted, whereas human infection has been documented for only certain genotypes [4]. This molecular classification has substantially improved our understanding of host specificity, transmission dynamics and the public health significance of different *E. bieneusi* genotypes [4,5].

Cattle are recognized as one of the principal reservoirs of *E. bieneusi* [7,9,10,11]. Besides harboring numerous host-adapted genotypes, bovine populations also carry several zoonotic genotypes that have been reported in humans, highlighting their epidemiological importance [3,4]. To date, at least 97 *E. bieneusi* genotypes have been identified in bovids across published studies [1,4].

Over the past two decades, molecular surveys have documented *E. bieneusi* infections in cattle from all inhabited continents. Two recent systematic reviews and meta-analyses estimated the global prevalence of bovine *E. bieneusi* infection at approximately 13–14%, although considerable variation exists among geographical regions, production systems and age categories [12,13]. Young calves consistently show the highest infection rates, while dairy cattle are generally more frequently infected than beef cattle [13]. The dominant bovine genotypes worldwide include J, I, BEB4 and BEB6, although zoonotic genotypes such as D, EbpC and Type IV have also been repeatedly identified, emphasizing the potential role of cattle in zoonotic transmission [4,12,13]. Recent large-scale studies have continued to identify novel genotypes and expand our understanding of the genetic diversity of *E. bieneusi* in cattle populations.

Despite increasing global attention, molecular epidemiological data from Europe remain relatively limited [14]. To date, only a limited number of studies have investigated *E. bieneusi* in cattle from European countries, including Germany, the Czech Republic, Portugal, Slovakia, Austria and Türkiye [15,16,17,18,19,20,21,22,23]. These studies have demonstrated the circulation of both host-adapted and zoonotic genotypes, while recent investigations in Türkiye additionally revealed extensive genetic diversity and environmental contamination within dairy farms [22].

However, no molecular study has previously investigated *E. bieneusi* in cattle from Romania, despite the importance of the dairy sector in Southeastern Europe. Establishing baseline epidemiological and molecular data is therefore essential for understanding the occurrence, genetic diversity and zoonotic potential of *E. bieneusi* in Romanian cattle populations.

Therefore, the aim of the present study was to determine, for the first time, the molecular prevalence and genetic diversity of *E. bieneusi* in dairy cattle from Romania using nested PCR amplification and sequence analysis of the ITS region of the rRNA gene, and to assess the phylogenetic relationships and potential zoonotic significance of the identified genotypes.

## 2. Materials and Methods

### 2.1. Fecal Specimen Collection

**Figure 1:**
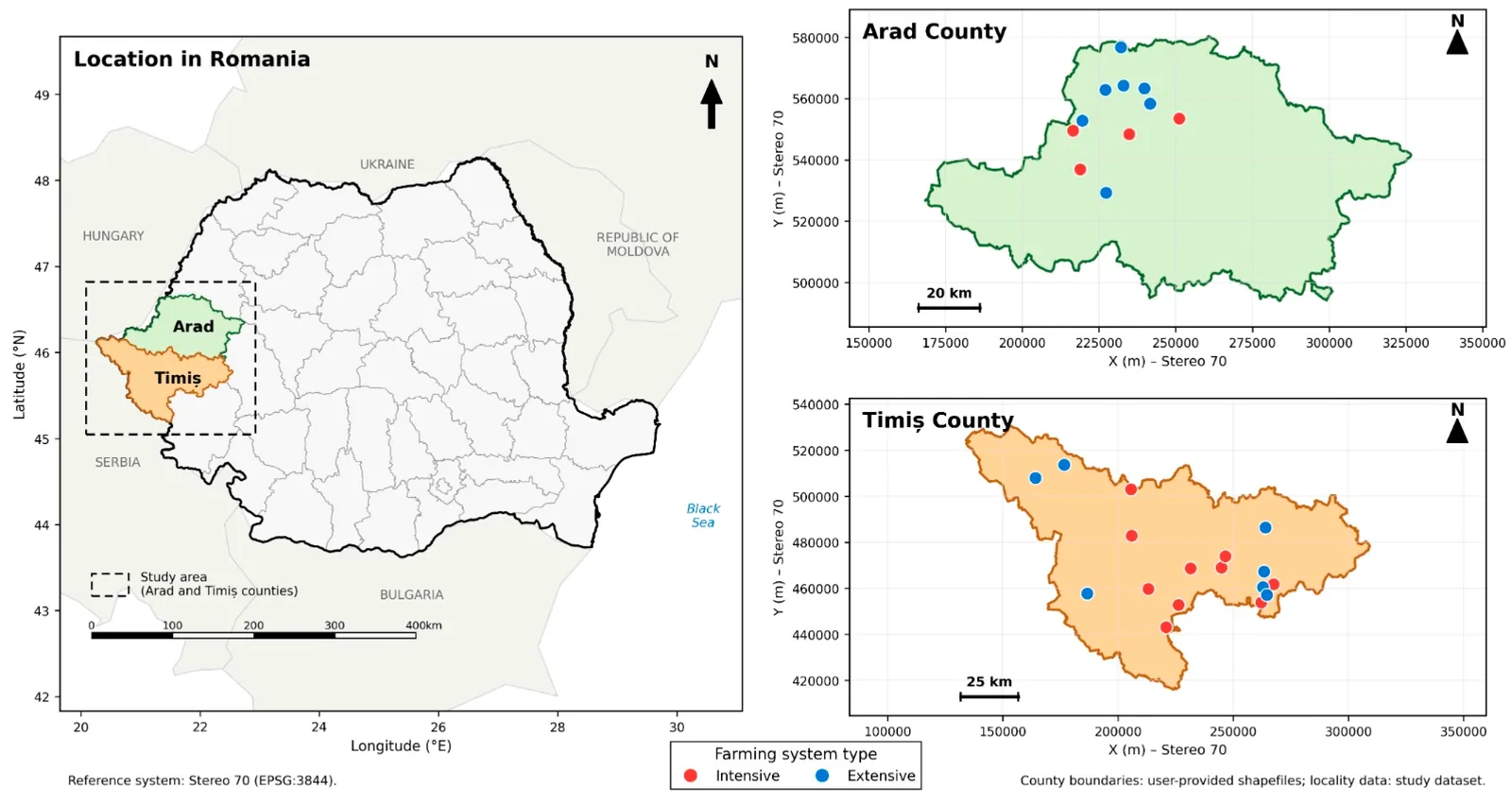
Geographical distribution of the study area in western Romania, showing the locations of Arad and Timiș counties and the investigated dairy farms. Red dots indicate farms operating under an intensive farming system, whereas blue dots indicate farms operating under an extensive farming system.

A cross-sectional baseline epidemiological survey was conducted between January and June 2026 to investigate the occurrence of *E. bieneusi* in dairy cattle from two counties located in western Romania (Arad and Timiș) (Figure 1). Given the absence of previous epidemiological data on *E. bieneusi* in Romanian cattle, the present study was designed to generate an initial regional molecular prevalence estimate and baseline epidemiological information for future nationwide investigations.

The minimum required sample size was calculated using Cochran’s formula for estimating prevalence in cross-sectional studies, assuming an expected *E. bieneusi* prevalence of 14.4%, based on the global meta-analysis of Qin et al. [13], a 95% confidence level (Z = 1.96), and a desired precision of 5% [24]. The calculated minimum sample size was 190 animals. Accordingly, fecal specimens were collected from a total of 190 dairy cattle originating from 28 dairy farms located in Arad (*n* = 11) and Timiș (*n* = 17) counties (Figure 1). Although the sample size was primarily determined to estimate the molecular prevalence of *E. bieneusi*, demographic, clinical, and management-related data were simultaneously collected to explore potential epidemiological associations and to establish a baseline dataset for future large-scale investigations.

Approximately 15–20 g of freshly voided feces was collected from each animal immediately after defecation using disposable sterile latex gloves and transferred into individually labeled sterile plastic containers. To avoid duplicate sampling, each animal was sampled only once during the study period. Farm managers voluntarily agreed to participate in the study and provided informed consent for sample collection. The sampling protocol was reviewed and approved by the Bioethics Committee of the University of Life Sciences “King Mihai I” from Timișoara (approval No. 589/5 September 2025). Following collection, all fecal specimens were stored under refrigerated conditions (<4 °C), transported to the Parasitology Laboratory of the Faculty of Veterinary Medicine, University of Life Sciences “King Mihai I” from Timișoara, and processed for molecular analysis as soon as possible.

At the time of sampling, epidemiological information was recorded for each animal using a standardized data collection form. The investigated variables included county of origin (Arad or Timiș), age category, sex, farming system, presence of diarrhea, and recent antimicrobial treatment history. Age was classified into pre-weaned calves (approximately up to 6–10 weeks of age, depending on farm management practices) and post-weaned cattle (completely transitioned to a solid-feed diet and not older than 12 months of age) [25]. The weaning status of each animal was determined based on its feeding status and the information provided by the respective farm regarding the age at weaning. Thus, the study population comprised young dairy cattle, including pre-weaned calves and post-weaned animals up to 12 months of age. Sex was categorized as male or female. In the investigated area, farms were categorized as intensive (>100 cattle) or extensive (≤20–30 cattle). No farms with an intermediate herd size of approximately 31–99 cattle were included in the study. The presence or absence of diarrhea was determined by a veterinarian at the time of sampling through visual assessment of fecal consistency, with liquid or semi-liquid feces classified as diarrheic. Recent antimicrobial treatment was defined as the administration of at least one systemic antimicrobial agent within the 28 days prior to sampling, as documented in farm treatment records and/or reported by the farm manager. Detailed information regarding the distribution of animals according to these epidemiological variables is presented in Table 1.

### 2.2. DNA Extraction and Molecular Detection of E. bieneusi

All fecal specimens were processed for molecular analyses within 48 h after collection. Genomic DNA was extracted from approximately 200 mg of each fecal sample using the Isolate Genomic DNA Kit (Bioline Reagents Limited, London, UK), following the manufacturer’s recommendations. Purified DNA was eluted in 100 μL of elution buffer and stored at −20 °C until molecular analysis.

The presence of *E. bieneusi* was investigated by nested polymerase chain reaction (PCR) amplification of the internal transcribed spacer (ITS) region of the ribosomal RNA (rRNA) gene, yielding an expected amplicon of approximately 392 bp. The assay was performed according to the protocol of Buckholt et al. [26], with minor modifications. The first-round PCR employed primers EB-F1 (5′-GGTCATAGGGATGAAGAG-3′) and EB-R1 (5′-TTCGAGTTCTTTCGCGCTC-3′), whereas the nested reaction used primers EB-F2 (5′-GCTCTGAATATCTATGGCT-3′) and EB-R2 (5′-ATCGCCGACGGATCCAAGTG-3′)

Both amplification reactions were carried out in a final volume of 25 μL containing 12.5 μL of MyTaq™ Red Mix (Bioline Reagents Limited, London, UK), 1 μL of each primer (10 pmol/μL), 1 μL of genomic DNA (primary PCR) or 1 μL of the first-round PCR product (secondary PCR), and nuclease-free water to complete the reaction volume. Amplifications were performed using a MyCycler™ Thermal Cycler (Bio-Rad Laboratories, Hercules, CA, USA) under the following conditions: initial denaturation at 95 °C for 1 min, followed by 35 cycles consisting of denaturation at 94 °C for 30 s, annealing at 57 °C for 30 s, and extension at 72 °C for 40 s, with a final extension step at 72 °C for 10 min.

Each PCR run included a positive control consisting of DNA extracted from a dog-derived *E. bieneusi* isolate previously confirmed by sequencing (GenBank accession number: PP983236), together with a negative control containing nuclease-free water. Amplification products were separated by electrophoresis on 2% agarose gels stained with RedSafe™ nucleic acid stain (iNtRON Biotechnology, Seongnam-si, Gyeonggi-do, Republic of Korea) and visualized under ultraviolet illumination. A 100 bp DNA ladder (Bioline Reagents Limited, London, UK) was used to estimate fragment size.

### 2.3. DNA Sequencing and Genotype Identification

All PCR-positive samples were subjected to bidirectional Sanger sequencing to determine *E. bieneusi* genotypes. Although all 27 PCR-positive samples were submitted for sequencing, only 20 yielded sequences of sufficient quality for reliable genotype assignment. Prior to sequencing, secondary PCR products were purified using the Isolate II PCR and Gel Kit (Bioline Reagents Limited, London, UK) according to the manufacturer’s instructions and submitted to a commercial sequencing facility (Macrogen Europe B.V., Amsterdam, The Netherlands). Chromatograms were visually inspected, edited, and assembled into consensus sequences using ChromasPro version 2.1.8 (Technelysium Pty Ltd., Tewantin, Australia). Genotype identification was performed by comparing the obtained ITS sequences with homologous sequences deposited in the GenBank database using the Basic Local Alignment Search Tool (BLAST (available online: https://blast.ncbi.nlm.nih.gov/Blast.cgi, accessed on 7 September 2026); National Center for Biotechnology Information, Bethesda, MD, USA). Sequences exhibiting identity with previously described isolates were assigned to established *E. bieneusi* genotypes according to the currently accepted nomenclature [7]. Sequence comparison showed that all isolates assigned to the same genotype were identical across the analyzed ITS region, with no intra-genotype nucleotide variation detected. Therefore, one representative sequence of each identified genotype was selected and deposited in the GenBank database under accession numbers PZ569993 (BEB4), PZ846585 (I), PZ846587 (J), PZ846586 (BEB17), and PZ860264 (BEB8).

### 2.4. Phylogenetic Analysis

Phylogenetic relationships among *E. bieneusi* sequences were inferred based on the internal transcribed spacer (ITS) region of the rRNA gene. To determine the phylogenetic position of the Romanian cattle isolates, the taxon selection proposed by Jiang et al. [8] was used and supplemented with cattle-derived sequences reported by Ladjal et al. [27] and the sequences generated in the present study. The final dataset comprised 117 *E. bieneusi* sequences representing all 15 currently recognized phylogenetic groups, with host species and geographical origin considered in the selection of representative sequences. Detailed information on the sequences included in the analysis is provided in Supplementary Table S1. The sequences were aligned using MAFFT [28]. Phylogenetic relationships were inferred using the Neighbor-Joining (NJ) method [29], with evolutionary distances calculated using the Maximum Composite Likelihood method and rate variation among sites modeled according to a gamma distribution. The NJ approach was selected as a distance-based method suitable for assessing the phylogenetic placement of the Romanian *E. bieneusi* sequences within the established phylogenetic groups, particularly given the comparative nature of the analysis and the use of ITS sequences from multiple host species and geographical origins. The robustness of the inferred topology was assessed by 1000 bootstrap replicates. The resulting tree was used primarily to evaluate the clustering and phylogenetic-group assignment of the Romanian isolates rather than to establish a comprehensive evolutionary model for *E. bieneusi*. All analyses were performed using MEGA12 [30]. In addition, a second phylogenetic analysis restricted to Groups 1, 2, 3, 8, and 14 was performed using 56 selected sequences; the corresponding taxon selection is indicated in Supplementary Table S1, and the resulting phylogenetic tree is presented in Supplementary Figure S1.

### 2.5. Statistical Analysis

Because multiple animals originated from the same farm, potential within-farm clustering was considered when interpreting the epidemiological associations. Given the exploratory nature of the study, the limited number of farms (*n* = 28) and PCR-positive animals (*n* = 27), no multivariable farm-level model was fitted. The individual-animal analyses were therefore retained as exploratory comparisons, and potential within-farm correlation was acknowledged as a limitation.

Statistical analyses were performed using IBM SPSS Statistics for Windows, version 29.0 (IBM Corp., Armonk, NY, USA). The prevalence of *E. bieneusi* infection was calculated as the proportion of PCR-positive animals among the total number of examined cattle, with corresponding 95% confidence intervals (CIs). Associations between *E. bieneusi* PCR positivity and the investigated epidemiological variables (county, age category, sex, farming system, presence of diarrhea, and recent antimicrobial treatment) were assessed using contingency-table analysis. Odds ratios (ORs) and their 95% CIs were calculated to estimate the strength of the associations. Fisher’s exact test was used when expected cell frequencies were low. A two-sided *p*-value < 0.05 was considered statistically significant. Given the relatively small number of PCR-positive animals, the association analysis was considered exploratory and was restricted to univariable comparisons.

## 3. Results

### 3.1. Molecular Prevalence of E. bieneusi

A total of 190 fecal samples collected from young dairy cattle on 28 farms in Arad (*n* = 11 farms) and Timiș (*n* = 17 farms) counties were examined by nested PCR targeting the ITS region of the *E. bieneusi* rRNA gene. Overall, *E. bieneusi* DNA was detected in 27 of 190 cattle (14.2%; 95% CI: 10.0–19.9%).

Positive animals were identified in both counties, with a prevalence of 14.3% (7/49; 95% CI: 7.1–26.7%) in Arad and 14.2% (20/141; 95% CI: 9.4–20.9%) in Timiș. No statistically significant difference in prevalence was observed between the two counties (OR = 1.01, 95% CI: 0.40–2.55; Fisher’s exact test, *p* = 1.000).

At the farm level, positive animals were identified on 10 of the 28 investigated farms (35.7%). Within positive farms, the proportion of PCR-positive cattle ranged from 14.3% to 50.0%. The highest farm-level prevalence was observed in one farm in Timiș County (50.0%; 2/4 animals), followed by two farms in Timiș (44.4%; 4/9 animals each). In contrast, no positive animals were detected on the remaining 18 farms.

### 3.2. Distribution of E. bieneusi Infection According to Epidemiological Characteristics

The distribution of *E. bieneusi*-positive cattle according to the investigated epidemiological variables is presented in Table 1.

Among pre-weaned cattle, 21 of 116 animals were PCR-positive (18.1%; 95% CI: 12.2–26.1%), compared with 6 of 74 post-weaned cattle (8.1%; 95% CI: 3.8–16.6%). Among pre-weaned cattle, 21 of 116 animals were PCR-positive (18.1%; 95% CI: 12.2–26.1%), compared with 6 of 74 post-weaned cattle (8.1%; 95% CI: 3.8–16.6%). Pre-weaned cattle had higher odds of *E. bieneusi* detection than post-weaned cattle (OR = 2.51, 95% CI: 0.96–6.54; Fisher’s exact test, *p* = 0.058).

The prevalence was 15.4% (18/117) among females and 12.3% (9/73) among males (OR = 0.77, 95% CI: 0.33–1.83; *p* = 0.671).

Similarly, *E. bieneusi* was detected in 17 of 122 cattle with diarrhea (13.9%) and in 10 of 68 cattle without diarrhea (14.7%), with no evidence of an association between diarrhea and PCR positivity (OR = 0.94, 95% CI: 0.40–2.18; *p* = 1.000).

Regarding farming system, 21 of 141 cattle from intensive farms were positive (14.9%) compared with 6 of 49 cattle from extensive farms (12.2%) (OR = 0.80, 95% CI: 0.30–2.11; *p* = 0.813).

Recent antimicrobial treatment was reported for 61 animals, of which 8 were PCR-positive (13.1%), compared with 19 positive animals among 129 cattle without recent antimicrobial treatment (14.7%). No significant association was observed between recent antimicrobial treatment and *E. bieneusi* detection (OR = 0.87, 95% CI: 0.36–2.13; *p* = 0.828).

### 3.3. Genotype Distribution

Of the 27 PCR-positive cattle, high-quality ITS sequences suitable for genotype identification were obtained from 20 animals (74.1%), whereas sequencing results from the remaining 7 animals (25.9%) were of insufficient quality for reliable genotype assignment.

Five established *E. bieneusi* genotypes were identified among the 20 successfully sequenced isolates: BEB4 (*n* = 9; 45.0%), I (*n* = 5; 25.0%), J (*n* = 2; 10.0%), BEB8 (*n* = 2; 10.0%) and BEB17 (*n* = 2; 10.0%) (Table 2). Genotype BEB4 was therefore the predominant genotype, accounting for nearly half of all successfully genotyped isolates. Genotypes I, J, BEB8 and BEB17 occurred less frequently.

The distribution of the identified genotypes according to selected epidemiological characteristics was also examined descriptively. Among the 17 successfully genotyped pre-weaned cattle, BEB4 was identified in eight animals, genotype I in five, J in two, and BEB8 and BEB17 in one animal each. Among the three successfully genotyped post-weaned cattle, one isolate each belonged to BEB4, BEB8, and BEB17. Regarding geographical origin, 17 genotyped isolates originated from Timiș County and three from Arad County. In Timiș, BEB4 (*n* = 7) was the most frequently identified genotype, followed by I (*n* = 5), J (*n* = 2), BEB8 (*n* = 2), and BEB17 (*n* = 1), whereas the three isolates from Arad comprised BEB4 (*n* = 2) and BEB17 (*n* = 1). Among cattle with diarrhea, the identified genotypes were BEB4 (*n* = 6), I (*n* = 4), BEB8 (*n* = 2), J (*n* = 1), and BEB17 (*n* = 1); among cattle without diarrhea, BEB4 (*n* = 3), I (*n* = 1), J (*n* = 1), and BEB17 (*n* = 1) were detected. At the farm level, the 20 genotyped isolates originated from nine farms. BEB4 occurred on five farms, genotype I on two farms, J on two farms, BEB8 on two farms, and BEB17 on two farms. Notably, four of the five genotype I isolates originated from a single farm in Timiș County. Given the limited number of successfully genotyped animals and the sparse distribution across categories, these observations are descriptive and no formal statistical comparison of genotype distribution was performed.

The seven PCR-positive samples for which genotype assignment was unsuccessful were distributed between Arad (*n* = 4) and Timiș (*n* = 3).

**Figure 2:**
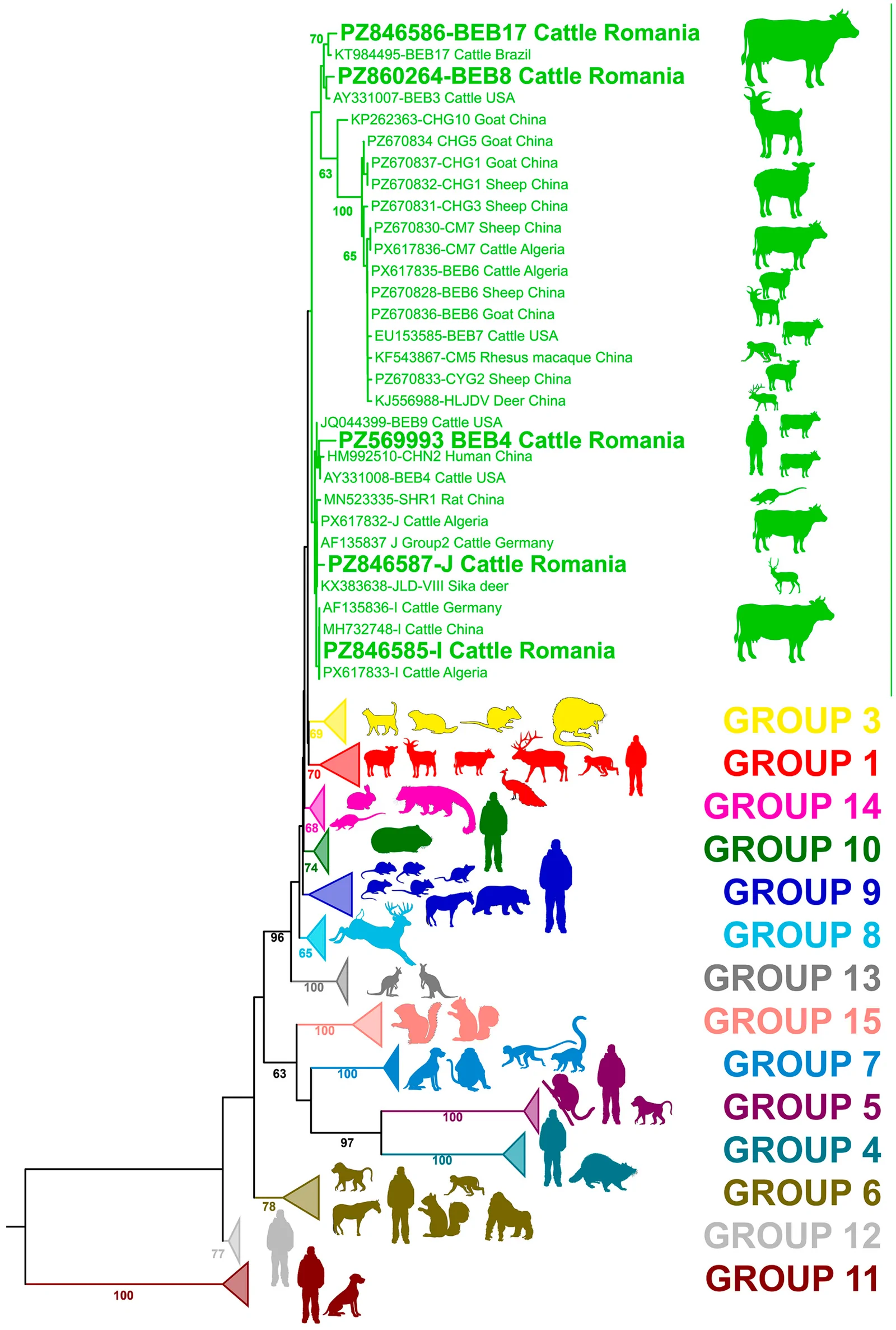
Phylogenetic relationships of *E. bieneusi* genotypes based on ITS sequences. The Neighbor-Joining tree was constructed using Maximum Composite Likelihood distances and 1000 bootstrap replicates. The analysis included 117 sequences representing all 15 recognized phylogenetic groups. Group 2 is shown in expanded form, whereas the remaining groups are collapsed for clarity. Romanian cattle-derived sequences are highlighted in bold green. All five Romanian genotypes (BEB17, BEB8, BEB4, J, and I) clustered within Group 2.

Phylogenetic analysis of ITS sequences placed all five Romanian *E. bieneusi* genotypes within phylogenetic Group 2 (Figure 2).

Phylogenetic analysis of ITS sequences placed all five Romanian *E. bieneusi* genotypes within phylogenetic Group 2 (Figure 2). The Romanian BEB17 sequence (PZ846586) grouped closely with a cattle-derived BEB17 sequence from Brazil, whereas BEB8 (PZ860264) was positioned within a broader ruminant-associated lineage containing other Group 2 genotypes. Genotype I (PZ846585) clustered with cattle-derived genotype I sequences reported from several geographical regions, while genotype J (PZ846587) occurred within the corresponding Group 2 lineage. The Romanian BEB4 sequence (PZ569993) was positioned within the BEB4-associated lineage containing cattle- and human-derived sequences. Overall, both the comprehensive and restricted analyses supported the placement of the Romanian isolates within Group 2.

## 4. Discussion

The present study provides the first molecular evidence of *E. bieneusi* infection in young dairy cattle from Romania, with an overall prevalence of 14.2% (27/190). This value is remarkably close to the global estimates reported by the two major systematic reviews and meta-analyses published in 2022. Thus, Qin et al. [13] reported an overall cattle prevalence of 12.9% (2566/19,791), whereas Taghipour et al. [12] estimated a pooled prevalence of approximately 14%. The prevalence observed in Romanian cattle is therefore within the range expected from the global epidemiological context and is particularly comparable with the pooled estimate reported for dairy cattle (14.4%) by Qin et al. [13].

The observed prevalence was also comparable with several molecular surveys conducted in European cattle populations, although considerable variation has been reported between countries. In the Czech Republic, Juránková et al. [17] detected *E. bieneusi* in 15.4% of cattle, while Rinder et al. [15] reported a prevalence of 10.7% (3/28) in the first molecular detection of the parasite in German cattle. More recently, Lichtmannsperger et al. [19] reported a lower prevalence of 4.6% (16/351) in Austrian cattle, including 7.9% among calves and 1.2% among adult cows. In Türkiye, prevalence estimates ranged from 12.8% (6/47) in a dairy farm [21] to 19.3% in healthy cattle [20], while a recent survey restricted to pre-weaned calves reported 15.8% (38/240) [23]. In contrast, a recent One Health survey in rural Adana found a substantially lower prevalence of 2.7% (2/75) in cattle [22]. Thus, the 14.2% prevalence observed in Romanian dairy cattle falls within the range reported in European molecular surveys and is particularly close to the estimates from the Czech Republic [17] and Türkiye [21,23].

The prevalence observed in Romanian dairy cattle was broadly comparable with molecular estimates reported from cattle populations worldwide. In Asia, Wang et al. [10] reported a prevalence of 13.0% among dairy cattle from different regions of China, while Del Coco et al. [31] detected *E. bieneusi* in 14.3% of dairy calves in Argentina, South America. In North America, Santín and Fayer [32] reported an overall prevalence of 24.0% in a longitudinal study of dairy cattle, whereas a lower prevalence of 10.4% was recorded among calves from multiple cattle farms in Australia [33]. In Africa, Ladjal et al. [27] recently reported a prevalence of 16.0% among cattle from Algeria. Overall, the 14.2% prevalence detected in Romanian dairy cattle is consistent with estimates reported across diverse cattle populations worldwide, although differences in age structure, production systems, geographical coverage and sampling design may account for the observed variation [12,13].

A particularly relevant finding of the present study was the higher prevalence among pre-weaned cattle (18.1%; 21/116) compared with post-weaned animals (8.1%; 6/74). However, this difference was not statistically significant (*p* = 0.058; OR = 2.51, 95% CI: 0.96–6.54). The observed direction of the association is consistent with the broader epidemiological evidence. Taghipour et al. [12] reported the highest pooled prevalence among calves, approximately 20%, while Qin et al. [13] found that cattle aged 3–12 months had a higher prevalence than older animals. Similarly, Juránková et al. [17] reported higher infection rates among calves up to three months of age than among older cattle, and Öncü Öner et al. [21] detected *E. bieneusi* in 19.2% of cattle younger than two months compared with 10% among cattle older than two months. These observations are consistent with previous reports showing higher *E. bieneusi* prevalence in younger cattle, although the underlying reasons were not investigated in the present study [9]. Nevertheless, because the association was not statistically significant in the present study, age should not be considered a confirmed risk factor based on the current dataset.

The absence of a statistically significant association between *E. bieneusi* infection and diarrhea is also noteworthy. The prevalence was similar among cattle with and without diarrhea, suggesting that detection of *E. bieneusi* DNA was not necessarily accompanied by overt gastrointestinal disease in the investigated population. This observation is compatible with the frequently reported asymptomatic nature of *E. bieneusi* infection in immunocompetent hosts and with the multifactorial etiology of diarrhea in cattle [4]. In young animals, diarrhea may result from a variety of infectious and non-infectious causes, and detection of *E. bieneusi* does not necessarily establish a causal relationship with clinical signs [17]. Similar findings were reported by Ladjal et al. [27], who did not identify a significant association between *E. bieneusi* infection and diarrhea in Algerian cattle.

No significant associations were observed between *E. bieneusi* positivity and sex, farming system or recent antimicrobial treatment. The absence of statistically significant relationships should, however, be interpreted cautiously because the relatively small number of positive animals limits the statistical power of the present study to detect modest associations. Moreover, recent antimicrobial treatment may be subject to confounding by indication, as treated animals may differ systematically from untreated animals because of underlying disease or management conditions, while the limited number of positive animals further reduced the statistical power to detect an association. Interestingly, the Austrian study reported higher infection rates among calves with a history of antimicrobial treatment [19], whereas no such association was detected in the present Romanian population. Differences in study populations, definitions of antimicrobial exposure and management practices may contribute to these contrasting observations.

Five established *E. bieneusi* genotypes were identified among the 20 successfully genotyped isolates: BEB4, I, J, BEB8 and BEB17. BEB4 was the predominant genotype, accounting for 45.0% of successfully genotyped isolates, followed by genotype I (25.0%), while J, BEB8 and BEB17 each accounted for 10.0%. The predominance of BEB4 and the presence of I and J are consistent with the established molecular epidemiology of *E. bieneusi* in cattle. Both systematic reviews [12,13] identified BEB4, I and J among the most frequently reported genotypes in bovine populations, although their relative frequencies vary substantially between geographical regions.

The genotype profile observed in Romania is particularly interesting in the European context. Four of the five genotypes identified in the present study, namely, I, J, BEB4 and BEB8, have also been reported in Austrian cattle. Lichtmannsperger et al. [19] identified I as the predominant genotype, followed by BEB4, J and BEB8, whereas BEB4 was the predominant genotype in the Romanian cattle examined here. The recurrence of these four genotypes in cattle from two geographically distinct European countries suggests that they may be relatively widespread components of the bovine *E. bieneusi* population in Europe [19], although this observation warrants further confirmation through multi-country studies. This overlap is particularly notable given the limited number of molecular studies available from European cattle populations.

The presence of genotypes I and J in Romanian cattle is also consistent with findings from the Czech Republic and Türkiye. Juránková et al. [17] identified genotype I in cattle from Czech herds, while the recent Turkish study of pre-weaned calves reported J and BEB8 among the identified genotypes [23]. In another Turkish dairy farm investigation, *E. bieneusi* was detected in 12.8% of cattle and several established and novel genotypes were identified [21]. The repeated detection of I, J, BEB4 and BEB8 across different European cattle populations suggests a degree of continuity in the bovine genotype pool, although local differences in genotype frequency remain evident [17,21,23].

The comparison with the recent Algerian investigation further illustrates the geographical variability in *E. bieneusi* genotype distributions. Ladjal et al. [27] reported a prevalence of 16.0% in cattle and identified both bovine-associated and genotypes with recognized zoonotic occurrence, whereas the genotypes detected in the present Romanian population were BEB4, I, J, BEB8 and BEB17. Although these differences may reflect geographical variation, they may also be influenced by differences in host composition, sample size and the proportion of positive samples successfully genotyped. Nevertheless, the contrasting genotype profiles emphasize the importance of generating country-specific molecular data rather than extrapolating the genotype distribution observed in one region to another.

The successful sequencing rate in the present study was 74.1%, with 20 of the 27 PCR-positive samples yielding sequences suitable for genotype assignment. The remaining seven positive samples could not be reliably genotyped, possibly because of insufficient DNA quantity or quality or other sequencing-related limitations. Likewise, although insufficient DNA quantity or quality may have contributed to the unsuccessful sequencing results, PCR inhibition cannot be completely excluded because replicate testing and a specific internal amplification control were not performed. Therefore, the reported genotype distribution represents only the successfully characterized subset and may not fully reflect the diversity among all infected cattle. Although selection bias cannot be excluded, five distinct genotypes were identified among the 20 successfully sequenced isolates, providing initial evidence of genetic diversity of *E. bieneusi* in young dairy cattle in western Romania. Similar difficulties in obtaining complete genotype information have been reported in other molecular surveys, particularly when parasite DNA concentrations are low or when amplification products do not yield high-quality sequences [21]. Nevertheless, the identification of five distinct genotypes among only 20 successfully sequenced isolates demonstrates considerable genotype diversity within the investigated Romanian cattle population.

Phylogenetic analysis further supported the genotype assignments and showed that all five Romanian *E. bieneusi* sequences belonged to phylogenetic Group 2, a lineage predominantly associated with ruminants but also known to contain genotypes reported from humans and other mammalian hosts [12,13]. The Romanian BEB17 sequence clustered closely with a cattle-derived BEB17 sequence from Brazil, while the BEB8 isolate was positioned within a broader ruminant-associated lineage containing bovine, ovine and caprine sequences. Likewise, the Romanian genotype I and J sequences grouped with homologous or closely related isolates previously identified in cattle from different geographical regions, consistent with the broad geographical distribution reported for these genotypes. The Romanian BEB4 sequence clustered within a lineage containing both cattle- and human-derived sequences. However, phylogenetic proximity alone does not demonstrate zoonotic transmission or establish cattle as a direct source of human infection. Therefore, the present findings should be interpreted as indicating potential zoonotic relevance rather than evidence of transmission, and further comparative molecular studies involving humans, animals, and environmental samples are required. Overall, the phylogenetic findings are consistent with the recognized genetic heterogeneity of Group 2 and further emphasize the importance of considering bovine *E. bieneusi* infections within a One Health context.

The present findings are particularly relevant in the context of the limited molecular evidence available from Europe. Compared with the extensive literature from China and other parts of Asia, relatively few studies have investigated *E. bieneusi* in European cattle. Molecular surveys have been conducted in countries including Germany, Portugal, the Czech Republic, Austria, Slovakia and Türkiye, but substantial geographical gaps remain. The present study therefore provides an important addition to the European epidemiological dataset by establishing the first molecular prevalence estimate for young dairy cattle in western Romania and documenting the circulation of five established genotypes [15,16,17,18,19,20,21].

Several limitations should be considered when interpreting the present findings. The geographical scope of sampling was limited to young dairy cattle from two counties in western Romania, and the study population comprised only pre-weaned calves and post-weaned cattle up to 12 months of age. Therefore, the findings should not be extrapolated directly to adult dairy cattle or considered representative of the entire Romanian dairy cattle population. The relatively small number of PCR-positive animals also limited the statistical power of the epidemiological analysis, particularly for detecting weaker associations. Furthermore, because animals were sampled within farms, potential within-farm clustering could not be fully accounted for in the exploratory individual-level analyses and may have affected the precision of the estimated associations. Moreover, seven PCR-positive samples could not be successfully genotyped, resulting in an incomplete assessment of the genotype distribution. An additional limitation of the present study is the potential clustering of observations within farms. Although 190 individual cattle from 28 farms were examined, the primary statistical comparisons were performed at the individual-animal level. Animals originating from the same farm may share management, environmental, and exposure conditions, resulting in within-farm correlation that could affect the precision of confidence intervals and association estimates. Given the relatively small number of farms and PCR-positive animals, the epidemiological analyses were considered exploratory, and the potential effect of farm-level clustering should be taken into account when interpreting these associations. Future studies involving larger numbers of farms should incorporate farm-level clustering explicitly into the statistical design and analysis. Finally, the cross-sectional design permits the estimation of prevalence and identification of associations but does not allow causal or temporal relationships to be established. Despite these limitations, the study provides a valuable baseline for future investigations involving larger cattle populations and broader geographical coverage across Romania.

## 5. Conclusions

The present study demonstrates that *E. bieneusi* is circulating among young dairy cattle in western Romania, with a molecular prevalence of 14.2%, comparable to estimates reported globally and in several European and other cattle populations. Although no investigated epidemiological factor showed a statistically significant association with infection, the higher prevalence observed among pre-weaned cattle was consistent with the age-related pattern reported in several previous studies. The identification of five established genotypes, particularly the predominance of BEB4 and the presence of I, J and BEB8, further expands the molecular epidemiological information available for European cattle. These findings establish an initial baseline for *E. bieneusi* surveillance in young dairy cattle in Romania and support the need for broader investigations encompassing adult dairy cattle, additional age groups and regions, animal populations and potential environmental sources within a One Health framework.

## Figures and Tables

**Table 1:** Distribution of *E. bieneusi* PCR-positive cattle according to epidemiological characteristics.

Variables	Category	Examined, *n*	Positive, *n*	Prevalence, %	OR (95% CI)	*p*-Value
County	Timiș	141	20	14.2	Reference	—
	Arad	49	7	14.3	1.01 (0.40–2.55)	1.000
Age	post-weaned	74	6	8.1	Reference	—
	pre-weaned	116	21	18.1	2.51 (0.96–6.54)	0.058
Sex	female	117	18	15.4	Reference	—
	male	73	9	12.3	0.77 (0.33–1.83)	0.671
Farming system	intensive	141	21	14.9	Reference	—
	extensive	49	6	12.2	0.80 (0.30–2.11)	0.813
Diarrhea	no	68	10	14.7	Reference	—
	yes	122	17	13.9	0.94 (0.40–2.18)	1.000
Recent antimicrobial treatment	no	129	19	14.7	Reference	—
	yes	61	8	13.1	0.87 (0.36–2.13)	0.828

Legend: OR, odds ratio; CI, confidence interval. *p*-values were calculated using Fisher’s exact test. The reference category is indicated where applicable. For each epidemiological variable, the total number of examined animals was 190.

**Table 2:** Distribution of *E. bieneusi* genotypes identified in Romanian dairy cattle.

Genotype	No. Isolates	% Among Genotyped Isolates (*n* = 20)	% Among PCR-Positive Cattle (*n* = 27)
BEB4	9	45.0	33.3
I	5	25.0	18.5
J	2	10.0	7.4
BEB8	2	10.0	7.4
BEB17	2	10.0	7.4
Successfully genotyped	20	100.0	74.1
Not genotyped	7	—	25.9
PCR-positive	27		100.0

## Data Availability

The data supporting the findings of this study are contained within the article and its Supplementary Materials. The ITS sequences generated in this study are available in GenBank under the accession numbers reported in the manuscript.

## Supplementary Materials

The following supporting information can be downloaded at https://www.mdpi.com/article/10.3390/ani16203178/s1. Figure S1: Phylogenetic relationships among 56 *E. bieneusi* ITS sequences representing Groups 1, 2, 3, 8, and 14; Table S1: *E. bieneusi* ITS sequences included in the phylogenetic analyses; Supplementary Dataset S1: Multiple-sequence alignment of the complete ITS dataset (117 sequences); Supplementary Data S1: Newick-formatted phylogenetic tree from the complete ITS dataset (117 sequences); Supplementary Dataset S2: Multiple–sequence alignment of the restricted ITS dataset (56 sequences; Groups 1, 2, 3, 8, and 14); Supplementary Data S2: Newick-formatted phylogenetic tree from the restricted ITS dataset (56 sequences; Groups 1, 2, 3, 8, and 14).
