# Supplementary material for: First Report of Molecular Prevalence and Genetic Characterization of Enterocytozoon bieneusi in Young Dairy Cattle in Western Romania
Source: Animals (Basel). 2026 Oct 10;16(20):3178. doi: 10.3390/ani16203178 (PMC13700560; doi:10.3390/ani16203178)
Supplement: Supplementary file 1 [file animals-16-03178-s001.zip › animals-4594673-supplementary.pdf]

Supporting information

First report of molecular prevalence and genetic characterization of *Enterocytozoon bieneusi* in Romanian dairy cattle

This Supporting Information contains the complete ITS multiple-sequence alignment and corresponding Newick-formatted tree used for the comprehensive phylogenetic analysis, the sequence metadata used for taxon selection, and the restricted analysis focusing on Groups 1, 2, 3, 8, and 14. The complete dataset comprises 117 sequences, whereas the restricted dataset comprises 56 sequences.

Contents

Supplementary Dataset S1. Multiple-sequence alignment of the complete ITS dataset (117 sequences)

- ✓ Supplementary Data S1. Newick-formatted phylogenetic tree from the complete ITS dataset (117 sequences)
- ✓ Table S1. *Enterocytozoon bieneusi* ITS sequences included in the phylogenetic analyses
- ✓ Supplementary Dataset S2. Multiple-sequence alignment of the restricted ITS dataset (56 sequences; Groups 1, 2, 3, 8, and 14)
- ✓ Supplementary Data S2. Newick-formatted phylogenetic tree from the restricted ITS dataset (56 sequences; Groups 1, 2, 3, 8, and 14)
- ✓ Figure S1. Restricted ITS phylogeny of Groups 1, 2, 3, 8, and 14

Supplementary Dataset S1. Multiple-sequence alignment of the complete ITS dataset (117 sequences)

Multiple-sequence alignment of the ITS region used for the comprehensive phylogenetic analysis representing all 15 recognized phylogenetic groups. The five Romanian cattle-derived sequences are included under GenBank accession numbers PZ569993 (BEB4), PZ846585 (I), PZ846587 (J), PZ846586 (BEB17), and PZ860264 (BEB8).

>AF101197_A_Human
ATTTTCAGTTTTTGG-GGTGTGGGTATCGGAATGTGTGGTAGGTGATGTGTGTGT-GTATGGGGGA-TGCCGAGGGGACCAGCGGTGCGGTGGTGTGTGTAGGCGTGAGAGTG-
TATCTGCAAGGGTGAGGGATGTGGGTGCAGTGAGTTAGAGGTGGTTCCATGTGGAATAGTGGGATTGGTACGTGATGGTTGGATGGGGGAATGATGTGTGTATGGGTGAGGAAAATCGGAGGTTGCN
>AF101200_D_Human
ATTTTCAGTTTTTGG-GGTGTGGGTATCGGAATGTGTGGTAGGTGATGTGTGTGT-GTATGGGGGA-TGCCGAGGGGACCAGCGGTGCGGTGGTGTGTGCAGGCGTGAGAGTG-
TATCTGCAAGTGTGAGGGATGTGGGTGCAGCGAGTTAGAGGTGGTTCCATGTGGAATAGTGGGATTGGTACGTGATGGTTGGATGGGGGAATGATGTGTGTATGGGTGAGGAAAATCGGAGGTTGCN
>AF135836_I_Cattle_Germany
ATTTTCAGTTTTTGG-GGTGTGGGTATCGGAATGTGTGATAGGTGATGTGTGTGT-GTATGGGGGA-TGCCGAGGGGACCAGCGGTGTGGTGGTGTGTGTAGGCGTGAGAGTG-
TATCTGTAAGGGTGAGGGATGTAGCTGCAGTGAGTTAGAGATGGTTCCATGAGGAATAGTGGGATTGGTACGTGATGGTTGGATGGGGGAATGATGTGTGTATGGGTGAGGAAAATCGGAGGTTGCN
>AF135837_J_Cattle_Germany
ATTTTCAGTTTTTGG-GGTGTGGGTATCGGAATGTGTGGTAGGTGATGTGTGTGT-GTATGGGGGA-TGCCGAGGGGACCAGCGGTGTGGTGGTGTGTGTAGGCGTGAGAGTG-
TATCTGTAAGGGTGAGGGATGTAGCTGCAGTGAGTTAGAGATGGTTCCATGAGGAATAGTGGGATTGGTACGTGATGGTTGGATGGGGGAATGATGTGTGTATGGGTGAGGAAAATCGGAGGTTGCN
>AY237209_WL1_Raccoon_USA
ATTTTCAGTTTTTCG-GGTGTTGGTATCGGAATGGATAAGAAATGATGTGTATGA-GGATGGAG--TCATATGTGAGAATAA-
GGTGTGTTGTTGTGTGTATGGATGATTAGTATATCTGTGAGGATGAGGGATGGTATTTTGTTAAGTTATTAGAGGAAGCATGTGAAATAAGTGGATGCGTATGTGATAGTTAGATAGTG-
AATGATGTGTGTATGGGTGATGAAAATTGGAGGTTGCN
>AY237210_WL2_Raccoon_USA
ATTTTCAGTTTTTCG-GGTGTTGGTATCGGAATGCATAAGAAATGATGTGTATGATGGATGGAG--TCATATGTGAGAATAA-GGTGTGTTGTTGTGT-
TATGGATGATTAGTATATCTGTGAGGATGAGGGATGGTATTTTGTTAAGTTATTAGAGGAAGCATGTGAAATAAGTGGATGTGTATGTGATAGTTAGATAGTG-
AATGATGTGTGTATGGGTGATGAAAATTGGAGGTTGCN
>AY237211_WL3_Raccoon_USA
ATTTTCAGTTTTTCG-GGTGTTGGTATCGGAATGCATAAGAAATGATGTGTATGATGGATGGAG--TCATATGTGAGAATAA-GGTGTGTTGTTGTGT-
TATGGATGATTAGTATATCTGTGAGGATGAGGGATGGTATTTTGTTAAGTTATTAGAGGAAGCATGTGAAATAAGTGGATGTGTATGTGATAGTTAGATAGTG-
AATGATGTGTGTATGGGTGATGAAAATTGGAGGTTACN
>AY237212_WL4_Muskrat_USA
ATTTTCAGTTTTTAG-GGTGTGGGTATCGGAATGTATTGTGAGTGATGTGTGTGT-ATA--GGGGA-TGCCGAGGGGACCAGCGGTGTGGTGGTATGTGTAGATGTGAGAGTG-
TATCTGTAAGGGTGAGGGATGTGGCTGCAGTGAGTTAGAGGTGGTTCCATGAGGAATAGTGGGATTGGTACGTGATGGTTGGATGGGAGAATGATGTGTGTATAGGTGAGGAAAATCGGAGGTTGCN
>AY237214_WL6_Muskrat_USA
ATTTTCAGTTTTTAG-GGTGTGGGTATCGGAATGTATTGTGAGTGATGTGTGTGT-ATA--GAGGA-TGCCGAGGGGACCAGCGGTGTGGTGGTATGTGTAGATGTGAGAGTG-
TATCTGTAAGGGTGAGGGATGTGGCTGCAGTGAGTTAGAGGTGGTTCCATGAGGAATAGTGGGATTGGTACGTGATGGTTGGATGGGGGAATGATGTGTGTATAGGTGAGGAAAATCGGAGGTTGCN
>AY331007_BEB3_Cattle_USA
ATTTTCAGTTTTTAG-GGTGTGAGTATCGGAATGTATAGTAGGTGATGTGTGTGT-GTATGGGGGA-TGCCGAGGGGACCAGCGGTGCGGTGGTGTGTGTAGGCGTGAGAGCG-
TATCTGTAAGTGTGAGGGATGTAGCTGCAGTGAGTTAGAGATGGTTCCATGAGGAATAGTGGGATTGGTACGTGATGGTTGGATGGGGGAATGATGTGTGTATGGGTGAGGAAAATCGGAGGTTGCN
>AY331008_BEB4_Cattle_USA
ATTTTCAGTTTTTGG-GGTGTGAGTATCGGAATGTGTAGTAGGTGATGTGTGTGT-GTATGGGGGA-TGCCGAGGGGACCAGCGGTGTGGTGGTGTGTGTAGGCGTGAGAGTG-
TATCTGTAAGGGTGAGGGATGTAGCTGCAGTGAGTTAGAGATGGTTCCATGAGGAATAGTGGGATTGGTACGTGATGGTTGGATGGGGGAATGATGTGTGTATGGGTGAGGAAAATCGGAGGTTGCN
>DQ683749_CAF4_Human_Gabon
NNNNTCAGTTTTTCATGG-GTTTATATCGGAATGCCGAGTGGATG-TGTGTGAGG-ATGGATGGTGTTGTGTGGGAGAAGAA-
GGGGTAGTGTTGTGTGTATGTGACCCTGTGATGTCTGGAATGAGGAGGGATGGTGTTGTGCCTGGTTATTGGAGGATCCATGTGAAACGAGGGGATGCCGAGGTGATGGTCAGATGGTG-
AATGATGTGTGTATGGGTGAGGAAAATTGGAGGTTGCN
>DQ885584_PtEb VIII_Cat_Portugal
ATTTTCAGTTTTTAG-GGTGTGGGTATCGGAATGTATTGTGAGTGATGTGTGTGT-ATA--GAGGA-TGCCGAGGGGACCAGCGGTGTGGTGGTACGTGTAGATGTGAGAGTG-
TATCTGTAAGGGTGAGGGATGTGGCTGCAGTGAGTTAGAGGTGGTTCCATGAGGAATAGTGGGGTTGGTACGTGATGGTTGGATGGGGGAATGATGTGTGTATAGGTGAGGAAAATCGGAGGTTGCN
>DQ885585_PtEb IX_Dog_Australia
ATTTCTAATTTTTTTGATGGTTTGT-TCGGAATGTAT-ATGTGTAATGTGATATTTATATATGGTGTTG--GAGGATCATATTGTTGTAGTAGTATATGT---TCTGAGA-TA-
TTTCAATTAAAGTAAGATGTGTAATTAGAGTGATTTAGAATTGATTGTTGTTAATATATAAATATTTGTATGT-ATGATGTGATAAAG-AATGTGATATGTATAGGATAAAAAAACGAGAAGTTGCN
>DQ885588_PtEb XII_Marmoset_Portugal
ATTTTCAGTTTTTCATGG-GTTTATATCGGAATGCCGAGTGGATG-TGTGTGAGG-ATGGATGGTGTTGTGTGGGAGAAGAA-
GGGGTAGTGTTGTGTGTATGTGACCCTGTGATGTCTGGAATGAGGAGGGATGGTGTTGTGCCTGGTTATTGGAGGATCCATGTGAAACGAGGGGATGCCGAGGTGATGGTCAGATGGTG-
AATGATGTGTGTATGGGTGAGGAAAATTGGAGGTTGCN
>EU153585_BEB7_Cattle_USA
ATTTTCAGTTTTTAG-GGTGTGAGTATCGGAATGTATAGTAGGTGATGTATGTGT-GTATGGGTGA-TGCCGAGGGGACCCGCTGTGCGGTGGTGTGTGTAGGCGTGAGAGTG-
TATCTGTAAGTGTGAGGGATGTAGCTGCAGTGAGTTAGAGATGGTTCCATGAGGAATAGTGGGATTGGTACACGATGGGTTGGATGGAGAATGATGTGTGTATGGGTGAGGAAAATCGGAGGTTGCN
>FJ439681_S5_Human_Malawi
ATTTTCAGTTTTTGG-GGTGTGGGTATCGGAATGTATGGTAGGTGATGTGTGTGT-GTATGGGGGA-TGCCGAGGGGACCAGCGGTGCGGTGGTGTGTGTAGGCGTGAGAGTG-
TATCTGCAAGGGTGAGGGATGTGGGTGCAGCGAGTTAGAGGTGGTTCCATGTGGAATAGTGGGATTGGTACGTGATGGTTGGATGGGGGAATGATGTGTGTATGGGTGAGGAAAATCGGAGGTTGCN
>FJ439683_S7_Human_Netherlands
ATTTTCAGTTTTTGG-GGTGTGGGTATCGGAATGTATTGTAGGTGATGTGTGTGT-GTATGGGGGA-TGCCGAGGAGACCAGCAGTATGGTGGTGTGTGTAGGTGTGAGAGTG-
TATCTGTAAGGGTGAGGGATGTGGCTGCAGTGAGTTAGAGGTGGTTCCATGGGGAATAGTGGGATTGGTACGTGATGGTTGGATGGGGGAATGATGTGTGTATGGGTGAGGAAAATCGGAGGTTGCN
>GQ406054_horse 2_Horse_Colombia
ATTTTCAGTTTTTA-GGGGGTGGATATCGGAATGTATTGTGGGGGATGTGTGTGT-GTATGAGG--TTGTAGAGGAGAAGAGTAGCGTGATGCTGTGTGTAGATGCGAGAGTG-
TATCCGTAAGCGTGGGGGATGTGGCCATAGTGAGTTAGAGGTGGTTCCATGTGGAATAGTGGGATTGGTACGTAATGGTTTGATGGGG-CATGGTGTGTGTATGGGTGAGAAAAATCGGAGGTTGCN
>HM992510_CHN2_Human_China
ATTTTCAGTTTTTGT-GGTGTGAGTATCGGAATGTGTAGTAGGTGATGTGTGTGT-GTATGGGGGA-TGCCGAGGGGACCAGCGGTGTGGTGGTGTGTGTAGGCGTGAGAGTG-
TATCTGTAAGGGTGAGGGATGTAGCTGCAGTGAGTTAGAGATGGTTCCATGAGGAATAGTGGGATTGGTACGTGATGGTTGGATGGGGGAATGATGTGTGTATGGGTGAGGAAAATCGGAGGTTGCN
>JF681179_KB5_Baboon_Kenya
ATTTTCAGTTTTTA-GGGGGTGGATATCGGAATGTATTGTGGGGGATGTGTGTGT-GTATGAGG--TTGTAGAGGAGAAGAGTAGCGTGATGCTGTGTGTAGATGTGAGAGTG-
TATCCGTAAGCGTGGGGGATGTGGCCATAGTGAGTTAGAGGTGGTTCCATGTGGAATAGTGGGATTGGTACGTAATGGTTTGATGGGG-CATGGTGTGTGTATGGGTGAGAAAAATCGGAGGTTGCN
>JF681180_KB6_Baboon_Kenya
ATTTTCAGTTTTTCATGG-GTTTATATCGGAATGCCAAGTGGATG-TGTGTGAGG-ATGGATGGTGTTGTGTGGGAGAAGAA-
GGGGTAGTGTTGTGTGTATGTGACCCTGTGATGTCTGGAATGAGGAGGGATGGCGTTGTGCCTGGTTATTGGAGGATCCATGTGAAACGAGGGGATGGCGAGGTGATGGTCAGATGGTG-
AATGATGTGTGTATGGGTGAGGAAAATTGGAGGTTGCN
>JN595887_17242259_Human
NNNT-CAGTTTTTA-GGGGGTGGATATCGGAATGTATCGTGGGGGATGCGTGTGT-GTGTGAGG--TTGTAGAGGAGAAGAGCAGCGTGATGCTGTGTGTAGATGTGAGAGTG-
TATCCGTAAGCGTGGGGGATGTGGCCATAGTGAGTTAGAGATGGTTCCATGGGGAATAGCGGGGTTGGTACGTGATGGGTGGATGGGG-AATGATGTGTGTATGGGTGAGGAAAATCGGAGGTTGCN
>JN997480_25005-Nig4_Human
ATTTTTAGTTTTTG-GGGTGTGGGTATCGGAATGTATCATAGATGATGTGTATGT-ATGTGGGG--T-GTAGAGGGGAAGAGTGGTGTGGTGGTATGTGTAGGTGTGAGAGTG-
TATCTGTAAGTGTGAGGAATGTGGCCATGGTGAGTTAGAGATGGTTCCATGTGGAATAGTGGGATTGGTACGTGATGGTTTGATGTGG-AATGGTATGTGTATGGGTGAGAAAAATCGGAGGTTGCN
>JQ044399_BEB9_Cattle_USA
ATTTTCAGTTTTTGG-GGTGTGGGTATCGGAATGTGTAGTAGGTGATGTGTGTGT-GTATGGGGGA-TGCCGAGGGGACCAGCGGTGTGGTGGTGTGTGTAGGCGTGAGAGTG-
TATCTGTAAGGGTGAGGGATGTAGCTGCAGTGAGTTAGAGATGGTTCCATGAGGAATAGTGGGATTGGTACGTGATGGTTGGATGGGGGAATGATGTGTGTATGGGTGAGGAAAATCGGAGGTTGCN
>JQ437575_KIN3_Human_Congo
ATTTTCAGTTTTTCATGG-GTTTATATCGGAATGTCGAGTGGGTG-GGTGTGAGG-ATGGATGGTGTTGTGTGGGAGAAGAA-
GGGGTAGTGTTGTGTGTATGTGACCCTGTGATGTCTGGAATGAGGAGGGATGGTGTTGTGCCTGGTTATTGGAGGATCCATGTGAAACGAGGGGATGGCGAGGTGATGGTCAGATGGTG-
AATGATGTGTGTATGGGTGAGGAAAATTGGAGGTTGCN
>JQ837797_gorilla3_Gorilla_Central_Africa
ATTT-TAGTTTTTA-GGGGGTGGATATCGGAATGTATCGTGGGGGATGCGTGTGT-GTGTGAGG--TTGTAGAGGAGAAGAGCAGCGTGATGCTGTGTGTAGATGTGAGAGTG-
TATCCGTAAGCGTGGGGGATGTGGCCATAGTGAGTTAGAGATGGTTCCATGGGGAATAGCGGGGTTGGTATGTGATGGGTGGATGGGG-AATGATGTGTGTATGGGTGAGGAAAATCGGAGGTTGCN
>JQ863269_WW1_Human_China
ATTTTCAGTTTTTAG-GGTGTGGGTATCGGAATGTATGGTAGATGATGTATGTGT-GTATGGGGGA-TGCCGAGGGGATCAGCGGTGTGGTGGTGTGTGTAGGCGTGAGAGTG-
TATCTGTAAGGGTGAGGGATGTGGGTGCAGTGAGTTAGAGGTGGTTCCATGTGGAATAGTGGGATTGGTACGTGATGATTGGGTGGGGGAATGATGTGTGTATGGGTGAGGAAAATCGGAGGTTGCN

>JQ863270_WW2_Human_China
ATTTTCAGTTTTTAG-GGTGTGGGTATCGGAATGTATGGTAGGTGATGTATGTGT-GTATGGGGGA-TGCCAAGGGGATCAGCGGTGTGGTGGTGTGTGTAGGCGTGAGAGTG-
TATCTGTAAGGGTGAGGGATGTGGGTGCAGTGAGTTAGAGGTGGTTCCATGTGGAATAGTGGGATTGGTACGTGATGATTGGATGGGGGAATGATGTGTGTATGGGTGAGGAAAATCGGAGGTTGCN
>JQ863271_WW3_Human_China
ATTTTCAGTTTTTAG-GACGTGAGTATCGGAATGTATTGTAGGTGATGTATGTGT-GTATGGGGGA-TGCCAAGGGGATCAGCGGTGTGGTGGTGTGTGTAGGCGTGAGAGTG-
TATCTGTAAGGGTGAGGGATGTGGGTGCAGTGAGTTAGAGGTGGTTCCATGTGGAATAGTGGGATTGGTACGTGATGATTGGATGGGGGAATGATGTGTGTATGGGTGAGGAAAATCGGAGGTTGCN
>JQ863272_WW4_Human_China
ATTTTCAGTTTTTAG-GACGTGAGTATCGGAATGTATTGTGGGAGATGTATGTGT-GTATGGGGGA-TGCCAAGGGGATCAGCGGTGTGGTGGTGTGTGTAGGCGTGAGAGTG-
TATCTGTAAGGGTGAGGGATGTGGGTGCAGTGAGTTAGAGGTGGTTCCATGTGGAATAGTGGGATTGGTACGTGATGATTGGATGGGGGAATGATGTGTGTATGGGTGAGGACAATCGGAGGTTGCN
>JQ863273_WW5_Human_China
ATTTTCAGCTTTTAG-GACGTGAGTATCGGAATGTATTGTAGGTGATGTATGTGT-GTATGGGGGA-TGCCAAGGGGATCAGCGGTGTGGTGGTGTGTGTAGGCGTGAGAGTG-
TATCTGTAAGGGCGAGGGATGTGGGGGCAGTGAGTTAGAGGTGGTTCCATGTGGAATAGTGGGATTGGTACGTGATGATTGGATGGGGGAATGATGTGTGTATGGGTGAGGAAAATCGGAGGTTGCN
>JQ863274_WW6_Human_China
ATTTTCAGTTTTTCG-GGTGTTGGTATCGGAATGGATAAGAAATGATGTGTATGC-GGATAGAG--TCATATGTGAGAATAA-
TGTGTGTTGTTGTATGTATGGATGATTAGTATATCTGTGAGGATGAGGGATGGTATTTTGTTAAGTTATTAGAGGAAGCATGTGAAATAAGTGGATGCGTATGTGATAGTTAGATAGTG-
AATGATGTGTGTATGGGTGAAGAAAATTGGAGGTTGCN
>JQ863275_WW7_Human_China
ATTTTCAGTTTTTAG-GGTGTGGGTATCGGAATGTATAAGAAATGATGTGTATGC-GGATAGAG--TCATATGTGAGAATAA-
TGTGTGTTGTTGTATGTATGGATGATTAGTGTATCTGTGAGGATGAGGGATGGTATTTTGTTAAGTTATTAGAGGAAGCATGTGAAATAAGTGGATGCGTATGTGATAGTTAGATAGTG-
AATGATGTGTGTATGGGTGAAGAAAATTGGAGGTTGCN
>JQ863276_WW8_Human_China
ATTTCTAATTTTTTTGATGGTTTGT-TCGGAATGCAT-ATGTGTAATGTGATATTTATATATGGTGTTG--GAGGATCATATTGTTGTAGTAGTATATGT---TCTGAGA-TA-
TTTCAATTAAAGTAAGATGTGTGATTAGAGTGATTTAGAATTGATTGTTGTTACAATATAAATACTTGTATGT-ATGATGTGATAAAG-AATGTGATATGTATAGGATAAAAAAACGAGAAGTTGCN
>JQ863277_19079_Wastewater
ATTTCTAATTTTTTTGATGGTTTGT-TCGGAATGTAT-ATGTGTAATGTGATATTTATATATGGTATTG--GAGGATCATATTGTTGTAGTAGTATATGT---TCTGAGA-TA-
TTTCAATTAAAGTAAGATGTGTAATTAGAGTGATTTAGAATTGATTGTTGTTAATATATAAATATTTGTATGT-ATGATGTGATAAAG-AATGTGATATGTATAGGATAAAAAAACGAGAAGTTGCN
>JX000572_Macaque1_Macaque_China
ATTTTCAGTTTTTTTGGGGGTGGACATCGGAATGTATTATGGGGGATGTGTGTGT-GTATGAGG--TTGTAGAGGAGAAGAGCAGCGTGATGCTATGTGTAGGTGTGAGAGTG-
TATCCGTAAACGTGAAGGATGTGGCCATAGTGAGTTAGAAGTGGTTCCATGTGGAATAGTGGGATTGGTACGTGATGGTTTGATGGGG-CATGGTGTGTGTATGGGTGAGAAAAATCGGAGGTTGCN
>JX524494_Nig7_Human_Nigeria
ATTTTTAGTTTTTG-GGGTGTGGGTGTCGGAATGTATCATAGATGATGTGTATGT-ATGTGGGG--TTGTAGAGGGGAAGAGTGGTGTGGTGGTATGTGTAGGTGTGAGAGTG-
TATCCGTAAGTGTGAGGAATGTGGCCATGGTGAGTTAGAGATGGTTCCATGTGGAATAGTGGGATTGGTACGTGATGGTTTGATGGGG-AATGATGTGTGTATGGGTGAGAAAAATCGGAGGTTGCN
>JX683806_31372_Human_Nigeria
ATTTTTAGTTTTTG-GGGTGTGGGTATCGGAATGTATCATAGATGATGTGTATGT-ATGTGGGG--TTGTAGAGGGGAAGAGTGGTGTGGTGGTATGTGTAGGTGTGAGAGTG-
TATCCGTAAGTGTGAGGAATGTGGCCATGGTGAGTTAGAGATGGTTCCATGTGGAATAGTGGGATTGGTACGTGATGGTTTGATGGGG-AATGATGTGTGTATGGGTGAGAAAAATCGGAGGTTGCN
>KF543866_CM4_Monkey_China
ATTTTCAGTTTTTAG-GGTGTGAGTATCGGAATATATCGTGAGTAATGTGCGTGT-GTGTGGGG--TTGTATAGGAGAGCAATGGTGTAGTGTTATGTGTAGGTGTGAGAGTG-
TATCCGTAAGCATGAGGGATGCGGTCTCAGCAAGTTACAGGTGGTTCCATGTGGAATAATTGGATGGGTATGTGATGGTTGTCCTGTG-AAGGATGTGTGTATGGGTGAGGAAAATTGGAAGTTGCN
>KF543867_CM5_Rhesus_macaque_China
ATTTTCAGTTTTTAG-GGTGTGAGTATCGGAATGTATAGTAGGTGATGTATGTGT-GTATGGGTGA-TGCCGAGGGGACCCGCGGTGCGGTGGTGTGTGTAGGCGTGATAGTG-
TATCTGTAAGTGTGAGGGATGTAGCTGCAGTGAGTTAGAGATGGTTCCATGAGGAATAGTGGGATTGGTACACGATGGGTTGGATGGAGAATGATGTGTGTATGGGTGAGGAAAATCGGAGGTTGCN
>KF591684_WL22_Woodchuck_USA
ATTTTCAGTTTTTAG-GGTGTGGGTATCGGAATGTGTGGTAGGTGATGTGTGTGT-GTATGGGGGA-TGCCGAGGGGACCAGCGGTGTGGTGGTATGTGTAGATGTGAGAGTG-
TATCTGTAAGGGTGAGGGATGTGGCTGCAGTGTGTTAGAGGTGGTTCCATGAGGAATAGTGGGATTGGTACGTGNTGGTTGGATGGGAGAATGATGTGTGTATAGGTGAGGAAAATCGGAGGTTGCN
>KF591685_VL23_Deer mouse_USA
ATTTTCAGTTTTTAG-GGTGTGGGTATCGGAATGTGTGGTAGGTGATGTGTGTGT-GTATGGGGGA-TGCCGAGGGGACCAGCGGTGTGGTGGTATGTGCAGACGTGAGAGTG-
TATCTGCAAGGGTGAGGGATGTGGCTGCAGCGAGTTAGAGGTGGTTCCATGAGGAATAGTGGGATTGGTACGTGATGGTTGGATGGGAGAATGATGTGTGTATAGGTGAGGAAAATCGGAGGTTGCN
>KF591686_WL25_Deer mouse_USA
ATTTTCAGTTTTTAG-GGTGTGGGTATCGGAATGTATTGTGAGTGATGTGTGTGT-ATA--GGGGA-TGCCGAGGGGACCAGCGGTGTGGTGGTATGTGTAGATGTGAGAGTG-
TATCTGTAAGGGTGAGGGATGTGGCTGCAGCGAGTTAGAGGTGGTTCCATGAGGAATAGTGGGATTGGTACGTGATGGTTGGATGGGAGAATGATGTGTGTATAGGTGAGGAAAATCGGAGGTTGCN
>KF591687_WL26_Racoon_USA
ATTTTCAGTTTTTCG-GGTGTTGGTATCGGAATGGATAAGAAATGATGTGTATGC-GGATGGAG--TCATATGTGAGAATAA-
TGTGTGTTGTTGTATGTATGGATGATTAGTATATCTGTGAGGATGAGGGATGGTATTTTGTTAAGTTATTAGAGGAAGCATGTGAAATAAGTGGATGCGTATGTGATAGTTAGATAGTG-
AATGATGTGTGTATGGGTGAAGAAAATTGGAGGTTGCN
>KJ556988_HLJDV_Deer_China
ATTTTCAGTTTTTAG-GGTGTGAGTATCGGAATGTATAGTAGGTGATGTATGTGT-GTATGGGTGA-TGCCGAGGGGACCCGCGGTGCGGTGGTGTGTGTAGGCGTGAGAGTG-
TATCTGTAAGTGTGAGGGATGTAGCTGCAGTGAGTTAAAGATGGTTCCATGAGGAATAGTGGGATTGGTACACGATGGGTTGGATGGAGAATGATGTGTGTATGGGTGAGGAAAATCGGAGGTTGCN
>KJ668732_CD5_Dog_China
ATTTTCAGTTTTTAG-GGTGTGAGTATCGGAATATATCGTGAGTAATGTGCGTGT-GTGTGGGG--TTGTATAGGAGAGCAATGGTGTAGTGTTATGTGTAGGTGTGAGAGTG-
TATCCGTAAGCATGAGGGATGCGGTCTCAGCAAGTTACGGGTGGTTCCATGTGGAATAATTGGATGGGTATGTGATGGTTGTCCTGTG-AAGGATGTGTGTATGGGTGAGGAAAATTGGAAGTTGCN
>KJ668734_CD7_Dog_China
ATTTCTAATTTTTTTGATGGTTTGT-TCGGAATGCAT-ATGTGTAATGTGATATTTATATATGGTGTTG--GAGGATCATATTGTTGTAGTAGTATATGT---TCTGAGA-TA-
TTTCAATTAAAGTAAGATGTGTAATTAGAGTGATTTAGAATTGATTGTTGTTAATATATAAATATTTGTATGT-ATGATGTGATAAAG-AATGTGATATGTATAGGATAAAAAAACGAGAAGTTGCN
>KJ668735_CD8_Dog_China
ATTTCTAATTTTTTTGATGGTTTGT-TCGGAATGCAT-ATGTGTAATGTGATATTTATATATGGTGTTG--GAGGATCATATTGTTGTAGTAGTATATGT---TCTGAGA-TA-
TTTCAATTAAAGTAAGATGTGTAATTAGAGTGATTTAGAATTGATTGTTGTTACAATATAAATACTTGTATGT-ATGATGTGATAAAG-AATGTGATATGTATAGGATAAAAAAACGAGAAGTTGCN
>KJ728811_CM18_Monkey_China
ATTTTCAGTTTTTAG-GGTGTGAGTATCGGAATATATCGTGAGTAATGTGCGTGT-GTGTGGGG--TTGTATAGGAGAGCAATGGTGTAGTGTTATGTGTAGGTGTGAGAGTG-
TATCCGTAAGCATGAGGGATGCGGTCTCAGCAAGTTACAGGTGGTTCCATGTGGAATAATTGGATGGGTATGTGATGGTTGCCCTGTG-AAGGATGTGTGTATGGGTGAGGAAAATTGGAAGTTGCN
>KJ867482_DeerEb3_white_tailed_deer_USA
ATTTTCAGTTTTTAG-GGTGTGTGTATCGGAGTGTATGACAGGTGATGTGTGTGT---ATGGGGGA-TGCTGAGGGGACCAGTGGTGTGGTGGTGTGTGTAGGCGTGAGAGTG-
TATCTGTAAGGGTGAGGGATGTGGCTGCAGTGAGTTAGAGGTGGTTCCATGGGGAATAGTGGGATTGGTACGTGATAGTTGGATAGGGGAATGATGTGTGTATGGGTGAGGAAAATCGGAGGTTGCN
>KJ867484_DeerEb5_white_tailed_deer_USA
ATTTTCAGTTTTTAG-GGTGTGTGTATCGGAATGTATGGTAGGTGATGTGTGTGT-GTATGGGGGA-TGCTGAGGGGACCAGTGGTGTGGTGGTGTGTGTAGGCGTGAGAGTG-
TATCTGTAAGGGTGAGGGATGTGGCTGCAGTGAGTTAGAGGTGGTTCCATGGGGAATAGTGGGATTGGTACGTGATAGTTGGATAGGGGAATGATGTGTGTATGGGTGAGGAAAATCGGAGGTTGCN
>KJ867485_DeerEb6_white_tailed_deer_USA
ATTTTCAGTTTTTAG-GGTGTGTGTATCGGAGTGTATGATAGGTGATGTGTGTGT---ATGGGGGA-TGCTGAGGGGACCAGTGGTGTGGTGGTGTGTGTAGGCGTGAGAGTG-
TATCTGTAAGGGTGAGGGATGTGGCTGCAGTGAGTTAGAGGTGGTTCCATGGGGAATAGTGGGATTGGTACGTGATAGTTGGATAGGGGAATGATGTGTGTATGGGTGAGGAAAATCGGAGGTTGCN
>KJ867487_DeerEb8_white_tailed_deer_USA
ATTTTCAGTTTTTAG-GGTGTGTGTATCGGAGTGTATGATAGGTGATGTGTGTGT-GTATGGGGGA-TGCTGAGGGGACCAGTGGTGTGGTGGTGTGTGTAGGCGTGAGAGTG-
TATCTGTAAGGGTGAGGGATGTAGCTGCAGTGAGTTAGAGGTGGTTCCATGGGGAATAGTGGGATTGGTACGTGATGGTTGGATGGGGGAATGATGTGTGGATGGGTGAGGAAAATCGGAGGTTGCN
>KJ867488_DeerEb9_white_tailed_deer_USA
ATTTTCAGTTTTTAG-GGTGTGTGTATCGGAATGTATGATAGGTGATGCGTGTGT-GTATGGGGGA-TGCTGAGGGGACCAGTGGTGTGGTGGTGTGTGTAGGCGTGAGAGTG-
TATCTGTAAGGGTGAGGGATGTAGCTGCAGTGAGTTAGAGGTGGTTCCATGGGGAATAGTGGGATTGGTACGTGATGGTTGGATGGGGGAATGATGTGTGTATGGGTGAGGAAAATCGGAGGTTGCN
>KM591951_XH3_Monkey_China
ATTTTCAGTTTTTAG-GGTGTGAGTATCGGAATATATCGTGAGTAATGTGCGTGT-GTGTGGGG--TTGTATAGGAGAGCAATGGTGTAGTGTTATGTGTAGGTATGAGAGTG-
TATCCGTAAGCATGAGGGATGCGGTTTCAGCAAGTTACAGGTGGTTCCATGTGGAATAATTGGATGGGTATGTGATGGTTGTCCTGTG-AAGGATGTGTGTATGGGTGAGGAAAATTGGAAGTTGCN
>KM870517_NED3_Dog_China
ATTTCTAATTTTTTTGATGGTTTGT-TCGGAATGTAT-ATGTGTAATGTGATATTTATATATGGTGTTG--GAGGATCATATTGTTGTAGTAGTATATGT---TCTGAGA-TA-
TTTTAATTAAAGTAAGATGTGTAATTAGAGTGATTTAGAATTGATTGTTGTTAATATATAAATATTTGTATGT-ATGATGTGATAAAG-AATGTGATATGTATAGGATAAAAAAACGAGAAGTTGCN
>KM870518_NED4_Dog_China
ATTTCTAATTTTTTTGATGGTTTGT-TCGGAATGTAT-ATGTGTAATGTGATATTTATATATGGTGTTG--GAGGATCATATTGTTGTAGTAGTATATGT---TCTGAGA-TA-
TTTCAATTAAAGTAAGATGTGTAATTAGAGTGATTTAGAATTGATTGTTGTTAATATATAAATATTTGTATGT-ATGATGTCATAAAG-AATGTGATATGTATAGGATAAAAAAACGAGAAGTTGCN
>KP262363_CHG10_Goat_China
ATTTTCAGTTTTTGG-GGTGTGGGTATCGGAATGTATGGTAGGTGATGTATGTGT-GTATGGGGGA-TGCCGAGGGGACCCGCGGTGCGGTGGTGTGTGTAGGCGTGAGAGTG-
TATCTGTAAGGGTGAGGGATGTGGGTGCAGCGAGTTAGAGATGGTTCCATGAGGAATAGTGGGATTGGTACACGATGGGTGGAAGGGAGAATGATGTGTGTATGGGTGAGGAAAATCGGAGGTTGCN
>KR062121_WR10_Bank_vole_Poland
ATTTTCAGTTTTTAG-GGTGTGGATATCGGAATGTATCGTAGATGATGTGTGTGT-GTGTGGGG-A-TGCCGAGGGGACCAGCGGTGTGGTAGTGTGTATAGACGTGAGAATG-
TATCTGTAAGGGTGAGGAATGTGGGTGCAGTGAGTTAGAGATGGTTCCATGTGGAATAGTGGGATTGGTACGTGATGGTTGGATGGGGGAATGATGTGTGTATGGGTGAGGAAAATCGGAGGTTGCN

>KR062123_WR9_Yellow_necked_mouse_Poland
ATTTTCAGTTTTTAG-GGTGTGGGTATCGGAATGTATCGCAGATGATGTATATGT-GTGTGGGG-A-TGCCGAGGGGACCAGCGGTGTGGTAGTGTGTATAGACGTGAGAATG-
TATCTGTAAGGGTGAGGAATGTGGGTGCAGTGAGTTAGAGATGGTTCCATGTGGAATAGTGGGATTGGTACGTGATGGTTGGATGGGGGAATGATGTGTGTATGGGTGAGGAAAATCGGAGGTTGCN
>KR062124_WR8_Striped_field_mouse_Poland
ATTTTCAGTTTTTAG-GGTGTGGGTATCGGAATGTATCGTAGATGATGTGTGTGT-GTGTGGGG-A-TGCCGAGGGGACCAGCGGTGTGGTAGTGTGTATAGGCATGAGAGTG-
TATCTGTAAGGGTGAGGAATGTGGGTGCAGTGAGTTAGAGATGGTTCCATGTGGAATAGTGGGATTGGTACGTGATGGTTGGATGGGGGAATGATGTGTGTATGGGTGAGGAAAATCGAAGGTTGCN
>KR062125_WR7_Striped_field_mouse_Poland
ATTTTCAGTTTTTAG-GGTGTGGATATCGGAATGTATCGTAGGTGATGTATATGT-GTATGGGGGA-TGCCGAGGGGACCAGCGGTGTAGTAGTATGTATAGGCGTGAGAGTG-
TATCTATAAGGGTGAGGAATGTGGGTGCAATGAGTTAGAGATGGTTCCATGTGGAATAGTGGGATTGGTACGTGATGGTTGGATAGGGGAATGATGTGTGTATAGGTGAGGAAAATCGGAGGTTGCN
>KT267286_CHK1_White_Kangaroo_China
ATTTTCAGTTTTTAG-GGTGTGGATATCGGAATGTATGGTAGGTGATGTGTGTAT-GTATGGGAGA-TGCCGAGGGAATCAGTGGTGTGGTGGTGTGTGTAGGTGTGAGAGTA-
TATTTGTAAGAGTGTGGGATGTGTCTGCAGTGAGTTATAGGTGGTTTCATGGGGAATAGTGGGATTGGTATGTGATGGTTGGATGGGGGAATGATGTGTGTATGGGTGAGGAAAATCGGAGGTTGCN
>KT267287_CHK2_Gray_Kangaroo_China
ATTTTCAGTTTTTAG-GGTGTGAATATCGGAATGTATGGTAGGTGATGTGTGTAT-GTATGGGAGA-TGCCGAGGGAATCAGTGGTGTGGTGGTGTGTGTAGGTGTGAGAGTA-
TATTTGTAAGAGTGTGGGATGTGTCTGCAGTGAGTTATAGGTGGTTTCATGGGGAATAGTGGGATTGGTATGTGATGGTTGGATGGGGGAATGATGTGTGTATGGGTGAGGAAAATCGGAGGTTGCN
>KT267293_CHB1_Black_Bear_China
ATTTTCAGTTTTTAG-GATGTGAGTATCGGAATGTATCGTAGGTGATGTATGTGT-GTGTGGGGGGGTGCCGAGGGGACCAGCGGTGTGGTGGTGTGTTTAAGTGTGAGAGTG-
TATCTGTAATGGTGAGTGATGTGGCTGCAGTGAGTTAGAGGTGGTTTCATGGGGAATAGTGGGATTGGTACGTGATGATTGGATGGGG-AATGATGTGTGTATGGGTGAGGAAAATCGGAGGTTGCN
>KT984495_BEB17_Cattle_Brazil
ATTTTCAGTTTTTGG-GGTGTGAGTATCGGAATGTGTAGTAGGTGATGTGTGTGT-GTATGGGGGA-TGCCGAGGGGACCCGCGGTGCGGTGGTGTGTGTAGGCGTGAGAGCG-
TATCTGTAAGTGTGAGGGATGTAGCTGCAGTGAGTTAGAGATGGTTCCATGAGGAATAGTGGGATTGGTACGTGATGGTTGGATGGGGGAATGATGTGTGTATGGGTGAGGAAAATCGGAGGTTGCN
>KU194603_XJH1_Horse_China
ATTTTCAGTTTTTA-GGGGGTGGATATCGGAATGTATTGTGAGGGATGTGTGTGT-GTATGAGG--TTGTAGAGGAGAAGAGTAGCGTGATGCTGTGTGTAGATGCGAGAGTG-
TATCCGTAAGCGTGGGGGATGTGGCCATAGTGAGTTAGAGGTGGTTCCATGTGGAATAGTGGGATTGGTACGTAATGGTTTGATGGGG-CATGGTGTGTGTATGGGTGAGAAAAATCGGAGGTTGCN
>KU194606_XJH4_Horse_China
ATTTTCAGTTTTTG-GGGTGTGGGTATCGGAATGTATGGTAGGTGATGTGTGTGT-GTATGAGG--TTGTAGAGGAGAAGAGTAGCGTGATGCTGTGTGTAGATGCGAGAGTG-
TATCCGTAAGCGTGGGGGATGTGGCCATAGTGAGTTAGAGGTGGTTCCATGTGGAATAGTGGGATTGGTACGTAATGGTTTGATGGGG-CATGGTGTGTGTATGGGTGAGAAAAATCGGAGGTTGCN
>KU847350_CE01_Squirrel_China
ATTTTCAGTTTTTA-GGGGGTGGATATCGGAATGTATTGTGGGGGATGTGTGTGT-GTATGAGG--TTGTAGAGGAGAAGAGTAGCGTGATGCTGTGTGTAGATGCGAGAGTG-
TATCCGTAAGCGTGGGGGATGTGGCCATAGTGAGTTAGAGGTGGTTCCATGTGGGATAGTGGGATTGGTACGTAATGGTTTGATGGGG-CATGGTGTGTGTATGGGTGAGAAAAATCGGAGGTTGCN
>KU885972_HNWW4_Human_China
ATTTTCAGTTTTTAG-GGTGTGGGTATCGGAATGTATTGTAGATGATGTATGTGT-GTATGGGGGA-TGCCGAGGGGATCAGCGGTGTGGTGGTGTGTGTAGGCGTGAGAGTG-
TATCTGTAAGGGTGAGGGATGTGGGTGCAGTGAGTTAGAGGTGGTTCCATGTGGAATAGTGGGATTGGTACGTGATGATTGGATGGGGGAATGATGTGTGTATGGGTGAGGAAAATCGGAGGTTGCN
>KX276711_SCH4_Horse_China
ATTTTCAGTTTTTAG-GATGTGAGTATCGGAATGTATCGTAGGTGATGTATGTGT-GTGTGGGGGGGTGCCGAGGGGACCAGCGGTGTGGTGGTGTGTTTAAGTGTGAGAGTG-
TATCTGTAATGGTGAGTGATGTGGCTGCAGTGAGTTAGAGGTGGTTTCATGGGGAATAGTGGGATTGGTACGTGATGATTGGATGGGG-AATGGTGTGTGTATGGGTGAGGAAAATCGGAGGTTGCN
>KX276712_YNH1_Horse_China
ATTTTCAGTTTTTA-GGGGGTGGATATCGGAATGTATTGTGGGGGATGTGTGTGT-GTATGAGG--TTGTAGAGGAGAAGAGTAGCGTGGTGCTGTGTGTAGATGCGAGAGTG-
TATCCGTAAGCGTGGGGGATGTGGCCATAGTGAGTTAGAGGTGGTTCCATGTGGAATAGTGGGATTGGTACGTAATGGTTTGATGGGG-CATGGTGTGTGTATGGGTGAGAAAAATCGGAGGTTGCN
>KX276714_YNH2_Horse_China
ATTTTCAGTTTTTA-GGGGGTGGATATCGGAGTGTATTGTGGGGGATGTGTGTGT-GTATGAGG--TTGTAGAGGAGAAGAGTAGCGTGATGCTGTGTGTAGATGCGAGAGTG-
TATCCGTAAGCGTGGGGGATGTGGCCATAGTGAGTTAGAGGTGGTTCCATGTGGAATAGTGGGATTGGTACGTAATGGTTTGATGGGG-CATGGTGTGTGTATGGGTGAGAAAAATCGGAGGTTGCN
>KX383638_JLD-VIII_Sika_deer_China
ATTTTCAGTTTTTGG-GGTGTGGGTATCGGAATGTGTGGTAGGTGATGTGTGTGT-GTATGGGGGA-TGCCGAGGGGACCAGCGGTGTGGTGGTGTGTGTAGGCGTGAGAGTG-
TATCTGTAAGGGTGAGGGATGTAGCTGCAGTGAGTTAGAGATGGTTCCATGAGGAATAGTGGGATTGGTACGTGATGGTTGGATGGGGGAATGATGTGTGTATGGGTGAGGAAAATCGGAGGTTGCN
>KY593707_EbRB_Human_Brazil
ATTTTCAGTTTTTGG-GGTGTGGGTATCGGAATGTATTGTAGGTGATGTATGTGT-GTATGGGGGA-TGCCGAGGGGACCAGCAGTGTGGTGGTGTGTGTAGGTGTGAGAGTG-
TATTTGTAAGGGTGAGGGATGTGGCTGCAGTGAGTTAGAGGTGGTTCCATGGGGAATAGTGGGATTGGTACGTGATGGTTGGATGGGGGAATGATGTGTGTATGGGTGAGGAAAATCGGAGGTTGCN
>KY706127_CSK1_White_Kangaroo_China
ATTTTCAGTTTTTAG-GGTGTGGATATCGGAATGTATGGTAGGTGATGTGTGTAT-GTATGGGAGA-TGCCGAGGGAATCAGTGGTGTGGTGGTGTGTGTAGGTGTGAGAGTA-
TATTTGTAAGAGTGTGGGATGTGTCTGCAGTGAGTTATAGGTGGTTTCATGGGGAATAGTGGGATTGGTATGTGATGGTTGGATGGGGGAATGATGTGTGTATGGGTGAGGAAAATCGGAGGTTGCN
>LC436503_WL2_deer_Korea
ATTTTCAGTTTTTGG-GGTGTGGGTATCGGAATGTGTGGTAGGTGATGTGTGTGT-GTATGGGGGA-TGCCGAGGGGACCAGCGGTGCGGTGGTGTGTGCAGGCGTGAGAGTG-
TATCTGCAAGTGTGAGGGATGTGGGTGCAGCGAGTTAGAGGTGGTTCCATGTGGAATAGTGTGATTGGTACGTGATGGTTGGATGGGGGAATGATGTGTGTATGGGTGAGGAAAATCGGAGGTTGCN
>MF410400_SCC1_Chipmunk_China
ATTTTCAGTTTTTTCGGGTGTGGGTATCGGAATGCATTGTGAATGATATGTGTGT-GTATGGGG--TTGTATAGGGGAACAATAGTGCGGTGTTGTGTGTAGGTGTGAGAATG-
TATCCGTAAGCATGGGGGATGTGGCTGCAGTGAGTTACAGGTGGTTCTATGCGGAACAATGAGATGGGTATGTGATGGTTGGATAGTG-AATGATGTGTGTATGGGTGAGGAAAATTGGAGATTGCN
>MF410401_SCC2_Chipmunk_China
ATTTTCAGTTTTTTCGGGGGTGGGTATCGGAATGCATTATGAATGATATGTGTGT-GTATGGGG--TTGTATAGGGGAACAATAGTGCGGTGTTGTGTGTAGGCGTGAGAATG-
TATCCGTAAGCATGGGGGATGTGGCTGCAGTGAGTTACAGGTGGTTCCATGCGGAACAATGAGATGGGTATGTGATGGTTGGATAGTG-AATGATGTGTGTATGGGTGAGGAAAATTGGAGATTGCN
>MF410402_SSCC3_Chipmunk_China
ATTTTCAGTTTTTTCGGGGGTGGGTATCGGAATGCATTATGAATGATATGTGTGT-GTATGGGG--TTGTATAGGGGAACAATAGTGCGGTGTTGTGTGTAGGTGTGAGAATG-
TATCCGTAAGCATGGGGGATGTGGCTGCAGTGAGTTACAGGTGGTTCCATGCGGAACAATGAGATGGGTATGTGATGGTTGGATAGTG-AATGATGTGTGTATGGGTGAGGAAAATTGGAGATTGCN
>MF410403_SCC4_Chipmunk_China
ATTTTCAGTTTTTTCGGGGGTGGGTATCGGAATGCATTATGAATGATATGTGTGT-GTATGGGG--TTGTATAGGGGAACAATAGTGCGGTGTTGTGTGTAGGTGTGAGAATG-
TATCCGTAAGCATGGGGGATGTGGCTGCAGTGAGTTACAGGTGGTTCCATGCGGAACAATGAGATGGGTATGCGATGGTTGGATAGTG-AATGATGTGTGTATGGGTGAGGAAAATTGGAGATTGCN
>MH732748_l_cattle_China
ATTTTCAGTTTTTGG-GGTGTGGGTATCGGAATGTGTGATAGGTGATGTGTGTGT-GTATGGGGGA-TGCCGAGGGGACCAGCGGTGTGGTGGTGTGTGTAGGCGTGAGAGTG-
TATCTGTAAGGGTGAGGGATGTAGCTGCAGTGAGTTAGAGATGGTTCCATGAGGAATAGTGGGATTGGTACGTGATGGTTGGATGGGGGAATGATGTGTGTATGGGTGAGGAAAATCGGAGGTTGCN
>MH940283_RS01_Squirrel_China
ATTTTCAGTTTTTTCGGGGGTGGGTATCGGAATGCATTATGAATGATATGTGTGT-GTATGGGG--TTGTATAGGGGAACAATAGTGCGGTGTTGTGTGTAGGCGTGAAAATG-
TATCCGTAAGCATGGGGGATGTGGCTGCAGTGAGTTACAGGTGGTTCCATGCGGAACAATGAGATGGGTATGTGATGGTTGGATAGTG-AATGATGTGTGTATGGGTGAGGAAAATTGGAGATTGCN
>MH940284_RS02_Squirrel_China
ATTTTCAGTTTTTTCGGGGGTGGGTATCAGAATGCTTTATGAATGATATGTGTGT-GTATGGGG--TTGTATAGGGGAACAATAGTGCGGTGTTGTGTGTAGGCGTGAAAATG-
TATCCGTAAGCATGGGGGATGTGGCTGCAGTGAGTTACAGGTGGTTCCATGCGGAACAATGAGATGGGTATGTGATGGTTGGATAGTG-AATGATGTGTGTATGGGTGAGGAAAATTGGAGATTGCN
>MK348513_MJ14_Binturong_China
ATTTTCAGTTTTTAG-GGTGTGGGTATCGGAATGCATGGTAGGTGATGTGTGTGT-GTATGGGGGA-TGCCGAGGGGACCAGCGGTGTGGTGGTGTGTGTAGGCGTGAGAGTG-
TATCTGTAAGGGTGAGGGATGTGGCTGCAGTGAGTTAGAGGTGGTTCCATGGGGAATAGTGGGATTGGTACGTGATGGTTGGATGGGGGAATGATGTGTGTATGGGTGAGGAAAATCGGAGGTTGCN
>MK809249_D_peacock_China
ATTTTCAGTTTTTGG-GGTGTGGGTATCGGAATGTGTGGTAGGTGATGTGTGTGT-GTATGGGGGA-TGCCGAGGGGACCAGCGGTGCGGTGGTGTGTGCAGGCGTGAGAGTG-
TATCTGCAAGTGTGAGGGATGTGGGTGCAGCGAGTTAGAGGTGGTTCCATGTGGAATAGTGGGATTGGTACGTGATGGTTGGATGGGGGAATGATGTGTGTATGGGTGAGGAAAATCGGAGGTTGCN
>MK909573_SCR06_Rabbit_China
ATTTTCAGTTTTTAG-GGTGTGGGTATCGGAATGCATGGTAGGTGATGTGTGTGT-GTATGGGGGA-TGCCGAGGGGACCAGCGGTGTGGTGGTGTGTGTAGGCGTGAGAGTG-
TATCTGTAAGGGTGAGGGATGTGGCTGCAGTGAGTTAGAGGTGGTTCCATGGGGAATAGTGGGATTGGTACGTGATGGTTGGATGGGGGAATGATGTGTGTATGGGTGAGGAAACTCGGAGGTTGCN
>MN523335_SHR1_Rat_China
ATTTTCAGTTTTTGG-GGTGTGGGTATCGGAATGTGTGGTAGGTGATGTGTGTGT-GTATGGGGGA-TGCCGAGGGGACCAGCGGTGTGGTGGTGTGTGTAGGTGTGAGAGTG-
TATCTGTAAGGGTGAGGGATGTAGCTGCAGTGAGTTAGAGATGGTTCCATGAGGAATAGTGGGATTGGTACGTGATGGTTGGATGGGGGAATGATGTGTGTATGGGTGAGGAAAATCGGAGGTTGCN
>MN523336_SHR1_Rat_China
ATTTTCAGTTTTTAG-GGTGTGGGTATCGGAATGCATGGTAGGTGATGTGTGTGT-GTATGGGGGA-TGCCGAGGGGACCAGCGGTGTGGTGGTGTGTGTAGGCGTGAGAGTG-
TATCTGTAAGGGTGAGGGATGTGGCTGCAGTGAGTTAGAGATGGTTCCATGGGGAATAGTGGGATTGGTACGTGATGGTTGGATGGGGGAATGATGTGTGTATGGGTGAGGAAAATCGGAGGTTGCN
>ON165751_XJHT4_Marmota_China
NNNNTCAGTTTTTTCGGGTGTGGGCATCGGAATGCATTATGAATGATATGTGTGT-GTATGGGG--TTGTATAGGGGAACAATAGTGCGGTGTTGTGTGTAGGTGTGAGAATG-
TATTCGTAAGCATGGGGGATGTGGCTGCAGTGAGTTACAGGTGGTTCCATGCGGAACAATGAGATGGGTATGTGATGGTTGGATAGTG-AATGATGTGTGTATGGGTGAGGAAAATTGGAGATTGCN
>OR838688_NegP1_Guinea_Pig_China
NNNNTCAGTTTTTGG-GGTGTGGGTATCGGAATGTATTGTAGGTAATGTGTGTGT-GTATGGGGGA-TGCCGAGGAGACCAGCAGTATGGTGGTGTGTGTAGGTGTGAGAGTG-
TATCTGTAAGGGTGAGGGATGTGGCTGCAGTGAGTTAGAGGTGGTTCCATGGGGAATAGTGGGATTGGTACGTGATGGTTGGATGGGGGAATGATGTGTGTATGGGTGAGGAAAATCGGAGGTTGCN
>OR838690_NESQ1_Squirrel_China
NNNNTCAGTTTTTTCGGGTGTGGGTATCGGAATGCATTATGAATGATATGTGTGT-GTATGGGG--TTGTATAGGGGAACAATAGTGCGGTGTTGTGTGTAGGCGTGAGAATG-
TATCCGTAAGCATGGGGGATGTGGCTGCAGTGAGTTACAGGTGGTTCCATGCGGAACAATGAGATGGGTATGTGATGGTTGGATAGTG-AATGATGTGTGTATGGGTGAGGAAAATTGGAGATTGCN

>PP905661_EBCMAP_019_Human_France
NNNNTCAGTTTTTGG-GGTGTGGGTATCGGAATGTATTGTAGGTGATGTATGTGT-GTATGGGGGA-TGCCGAGGGGACCAGCAGTGTGGTGGTGTGTGTAGGTGTGAGAGTG-
TATCTGTAAGGGTGAGGGATGTGGCTGCAGTGAGTTAGAGGTGGTTCCATGGGGAATAGTGGGATTGGTACATGATGGTTGGATGGGGAAATGATGTGTGTATGGGTGAGGAAAATCGGAGGTTGCN
>PV715457_Peru8_macaque_China
ATTTTCAGTTTTTGG-GGTGTGGGTATCGGAATGTGTGGTAGGTGATGTGTGTGT-GTATGGGGGA-TGCCGAGGGGACCAGCGGTGCGGTGGTGTGTGCAGGCGTGAGAGTG-
TATCTGCAAGGGTGAGGGATGTGGGTGCAGCGAGTTAGAGGTGGTTCCATGTGGAATAGTGGGATTGGTACGTGATGGTTGGATGGGGGAATGATGTGTGTATGGGTGAGGAAAATCGGAGGTTGCN
>PX617831_TypeIV_Cattle_Algeria
ATTTTCAGTTTTTGG-GGTGTGGGTATCGGAATGTGTGGTAGGTGATGTGTGTGT-GTATGGGGGA-TGCCGAGGGGACCAGCGGTGCGGTGGTGTGTGTAGGCGTGAGAGTG-
TATCTGCAAGGGTGAGGGATGTGGGTGCAGCGAGTTAGAGGTGGTTCCATGTGGAATAGTGGGATTGGTACGTGATGGTTGGATGGGGGAATGATGTGTGTATGGGTGAGGAAAATCGGAGGTTGCN
>PX617832_J_Cattle_Algeria
ATTTTCAGTTTTTGG-GGTGTGGGTATCGGAATGTGTGGTAGGTGATGTGTGTGT-GTATGGGGGA-TGCCGAGGGGACCAGCGGTGTGGTGGTGTGTGTAGGCGTGAGAGTG-
TATCTGTAAGGGTGAGGGATGTAGCTGCAGTGAGTTAGAGATGGTTCCATGAGGAATAGTGGGATTGGTACGTGATGGTTGGATGGGGGAATGATGTGTGTATGGGTGAGGAAAATCGGAGGTTGCN
>PX617833_I_Cattle_Algeria
ATTTTCAGTTTTTGG-GGTGTGGGTATCGGAATGTGTGATAGGTGATGTGTGTGT-GTATGGGGGA-TGCCGAGGGGACCAGCGGTGTGGTGGTGTGTGTAGGCGTGAGAGTG-
TATCTGTAAGGGTGAGGGATGTAGCTGCAGTGAGTTAGAGATGGTTCCATGAGGAATAGTGGGATTGGTACGTGATGGTTGGATGGGGGAATGATGTGTGTATGGGTGAGGAAAATCGGAGGTTGCN
>PX617834_D_Cattle_Algeria
ATTTTCAGTTTTTGG-GGTGTGGGTATCGGAATGTGTGGTAGGTGATGTGTGTGT-GTATGGGGGA-TGCCGAGGGGACCAGCGGTGCGGTGGTGTGTGCAGGCGTGAGAGTG-
TATCTGCAAGTGTGAGGGATGTGGGTGCAGCGAGTTAGAGGTGGTTCCATGTGGAATAGTGGGATTGGTACGTGATGGTTGGATGGGGGAATGATGTGTGTATGGGTGAGGAAAATCGGAGGTTGCN
>PX617835_BEB6_Cattle_Algeria
ATTTTCAGTTTTTAG-GGTGTGAGTATCGGAATGTATAGTAGGTGATGTATGTGT-GTATGGGTGA-TGCCGAGGGGACCCGCGGTGCGGTGGTGTGTGTAGGCGTGAGAGTG-
TATCTGTAAGTGTGAGGGATGTAGCTGCAGTGAGTTAGAGATGGTTCCATGAGGAATAGTGGGATTGGTACACGATGGGTTGGATGGAGAATGATGTGTGTATGGGTGAGGAAAATCGGAGGTTGCN
>PX617836_CM7_Cattle_Algeria
ATTTTCAGTTTTTAG-GGTGTGAGTATCGGAATGTATAGTAGGTGATGTATGTGT-GTATGGGTGA-TGCCGAGGGGACCCGTGGTGCGGTGGTGTGTGTAGGCGTGAGAGTG-
TATCTGTAAGTGTGAGGGATGTAGCTGCAGTGAGTTAGAGATGGTTCCATGAGGAATAGTGGGATTGGTACACGATGGGTTGGATGGAGAATGATGTGTGTATGGGTGAGGAAAATCGGAGGTTGCN
>PX617837_Peru6_Cattle_Algeria
ATTTTCAGTTTTTGG-GGTGTGGGTATCGGAATGTATGGTAGATGATGTGTGTGT-GTATGGGAGA-TGCCGAGGGGACCAGCGGTGCGGTGGTGTGTGTAGGCGTAAGAGTG-
TATCTGCAAGGGTGAGGGATGTGGGTGCAGCGAGTTAGAGGTGGTTCCATGTGGAATAGTGGGATTGGTACGTGATGGTTGGATGGGGGAATGATGTGTGTATGGGTGAGGAAAATCGGAGGTTGCN
>PZ670828_BEB6_sheep_China
ATTTTCAGTTTTTAG-GGTGTGAGTATCGGAATGTATAGTAGGTGATGTATGTGT-GTATGGGTGA-TGCCGAGGGGACCCGCGGTGCGGTGGTGTGTGTAGGCGTGAGAGTG-
TATCTGTAAGTGTGAGGGATGTAGCTGCAGTGAGTTAGAGATGGTTCCATGAGGAATAGTGGGATTGGTACACGATGGGTTGGATGGAGAATGATGTGTGTATGGGTGAGGAAAATCGGAGGTTGCN
>PZ670829_TypeIV_sheep_China
ATTTTCAGTTTTTGG-GGTGTGGGTATCGGAATGTGTGGTAGGTGATGTGTGTGT-GTATGGGGGA-TGCCGAGGGGACCAGCGGTGCGGTGGTGTGTGTAGGCGTGAGAGTG-
TATCTGCAAGGGTGAGGGATGTGGGTGCAGCGAGTTAGAGGTGGTTCCATGTGGAATAGTGGGATTGGTACGTGATGGTTGGATGGGGGAATGATGTGTGTATGGGTGAGGAAAATCGGAGGTTGCN
>PZ670830_CM7_sheep_China
ATTTTCAGTTTTTAG-GGTGTGAGTATCGGAATGTATAGTAGGTGATGTATGTGT-GTATGGGTGA-TGCCGAGGGGACCCGTGGTGCGGTGGTGTGTGTAGGCGTGAGAGTG-
TATCTGTAAGTGTGAGGGATGTAGCTGCAGTGAGTTAGAGATGGTTCCATGAGGAATAGTGGGATTGGTACACGATGGGTTGGATGGAGAATGATGTGTGTATGGGTGAGGAAAATCGGAGGTTGCN
>PZ670831_CHG3_sheep_China
ATTTTCAGTTTTTAG-GGTGTGAGTATCGGAATGTATAGTAGGTGATGTATGTGT-GTATGGGGGA-TGCCGAGGGGACCCGCGGTGCGGTGGTGTGTGTAGGCGTGAGAGCG-
TATCTGTAAGTGTGAGGGATGTAGCTGCAGTGAGTTAGAGATGGTTCCATGAGGAATAGTGGGATTGGTACACGATGGGTTGGATGGAGAATGATGTGTGTATGGGTGAGGAAAATCGGAGGTTGCN
>PZ670832_CHG1_sheep_China
ATTTTCAGTTTTTAG-GGTGTGAGTATCGGAATGTACAGTAGGTGATGTATGTGT-GTATGGGGGA-TGCCGAGGGGACCCGCGGTGCGGTGGTGTGTGTAGGCGTGAGAGTG-
TATCTGTAAGTGTGAGGGATGTAGCTGCAGTGAGTTAGAGATGGTTCCATGAGGAATAGTGGGATTGGTACACGATGGGTTGTATGGAGAATGATGTGTGTATGGGTGAGGAAAATCGGAGGTTGCN
>PZ670833_CYG2_sheep_China
ATTTTCAGTTTTTAG-GGTGTGAGTATCGGAATGTACAGTAGGTGATGTATGTGT-GTATGGGTGA-TGCCGAGGGGACCCGCGGTGCGGTGGTGTGTGTAGGCGTGAGAGTG-
TATCTGTAAGTGTGAGGGATGTAGCTGCAGTGAGTTAGAGATGGTTCCATGAGGAATAGTGGGATTGGTACACGATGGGTTGGATGGAGAATGATGTGTGTATGGGTGAGGAAAATCGGAGGTTGCN
>PZ670834_CHG5_goat_China
ATTTTCAGTTTTTAG-GGTGTGAGTATCGGAATGTATAGTAGGTGATGTATGTGT-GTATGGGGGA-TGCCGAGGGGACCCGCGGTGCGGTGGTGTGTGTAGGCGTGAGAGTG-
TATCTGTAAGTGTGAGGGATGTAGCTGCAGTGAGTTAGAGATGGTTCCATGAGGAATAGTGGGATTGGTACACGATGGGTTGTATGGAGAATGATGTGTGTATGGGTGAGGAAAATCGGAGGTTGCN
>PZ670835_Type_goat_China
ATTTTCAGTTTTTGG-GGTGTGGGTATCGGAATGTGTGGTAGGTGATGTGTGTGT-GTATGGGGGA-TGCCGAGGGGACCAGCGGTGCGGTGGTGTGTGTAGGCGTGAGAGTG-
TATCTGCAAGGGTGAGGGATGTGGGTGCAGCGAGTTAGAGGTGGTTCCATGTGGAATAGTGGGATTGGTACGTGATGGTTGGATGGGGGAATGATGTGTGTATGGGTGAGGAAAATCGGAGGTTGCN
>PZ670836_BEB6_goat_China
ATTTTCAGTTTTTAG-GGTGTGAGTATCGGAATGTATAGTAGGTGATGTATGTGT-GTATGGGTGA-TGCCGAGGGGACCCGCGGTGCGGTGGTGTGTGTAGGCGTGAGAGTG-
TATCTGTAAGTGTGAGGGATGTAGCTGCAGTGAGTTAGAGATGGTTCCATGAGGAATAGTGGGATTGGTACACGATGGGTTGGATGGAGAATGATGTGTGTATGGGTGAGGAAAATCGGAGGTTGCN
>PZ670837_CHG1_goat_China
ATTTTCAGTTTTTAG-GGTGTGAGTATCGGAATGTACAGTAGGTGATGTATGTGT-GTATGGGGGA-TGCCGAGGGGACCCGCGGTGCGGTGGTGTGTGTAGGCGTGAGAGTG-
TATCTGTAAGTGTGAGGGATGTAGCTGCAGTGAGTTAGAGATGGTTCCATGAGGAATAGTGGGATTGGTACACGATGGGTTGTATGGAGAATGATGTGTGTATGGGTGAGGAAAATCGGAGGTTGCN
>PZ846585_I_Cattle_Romania
ATTTTCAGTTTTTGG-GGTGTGGGTATCGGAATGTGTGATAGGTGATGTGTGTGT-GTATGGGGGA-TGCCGAGGGGACCAGCGGTGTGGTGGTGTGTGTAGGCGTGAGAGTG-
TATCTGTAAGGGTGAGGGATGTAGCTGCAGTGAGTTAGAGATGGTTCCATGAGGAATAGTGGGATTGGTACGTGATGGTTGGATGGGGGAATGATGTGTGTATGGGTGAGGAAAATCGGAGGTTGCN
>PZ846586_BEB17_Cattle_Romania
NNNTGCACTTTTTGG-GGTGTGAGTATCGGAATGTGTAGTAGGTGATGTGTGTGT-GTATGGGGGA-TGCCGAGGGGACCCGCGGTGCGGTGGTGTGTGTAGGCGTGAGAGCG-
TATCTGTAAGGGTGAGGGATGTAGCTGCAGTGAGTTAGAGATGGTTCCATGAGGAATAGTGGGATTGGTACGTGATGGTTGGATGGGGGAATGATGTGTGTATGGGTGAGGAAAATCGGAGGTTGCN
>PZ846587_J_Cattle_Romania
ATTTTCAGTTTTTGG-GGTGTGGGTATCGGAATGTGTGG-AGGTGATGTGTGTGT-GTATGGGGGA-TGCCGAGGGGACCAGCGGTGTGGTGGTGTGTGTAGGCGTGAGAGTG-
TATCTGTAAGGGTGAGGGATGTAGCTGCAGTGAGTTAGAGATGGTTCCATGAGGAATAGTGGGATTGGTACGTGATGGTTGGATGGGGGAATGATGTGTGTATGGGTGACGAAAATCGAAGGTTGCN
>PZ860264_BEB8_Cattle_Romania
NNNTTCAGTTTTTAGAGGTGTGGGTATCGGAATGTATAGTAGGTGATGTATGTGT-GTATGGGGGA-TGCCGAGGGGACCCGCGGTGCGGTGGTGTGTGTAGGCGTGAGAGCG-
TATCTGTAAGTGTGAGGGATGTAGCTGCAGTGAGTTAGAGATGGTTCCATGAGGAATAGTGGGATTGGTACGTGATGGTTGGATGGGGGAATGATGTGTGTATGGGTGAGGAAAATCGGAGGTTGCN
>PZ569993_BEB4_Cattle_Romania
AT-------------------------------------AAGGTGA-ATGTGTGTGTGAATGGGGGATGCCGAGGGGACCAGCGGTGTGGTGGTGTGTGTAGGCGTGAGAGTG-
TATCTGTAAGGGTGAGGGATGTAGCTGCAGTGAGTTAGAGATGGTTCCATGAGGAATAGTGGGATTGGTACGTGATGGTTGGATGGGGGAATGATGTGTGTATGGGTGAGGAAAATCGGAGGTTGCG

Supplementary Data S1. Newick-formatted phylogenetic tree from the complete ITS dataset (117 sequences)

Machine-readable Newick representation of the Neighbor-Joining tree inferred from the complete ITS dataset. Branch lengths represent evolutionary distances calculated using the Maximum Composite Likelihood method. Internal-node annotations retain the bootstrap-support values and MEGA12-exported standard-deviation fields obtained from 1,000 bootstrap replicates. Rate variation among sites was modeled using a gamma distribution.

((((((((((((((((((((((((EU153585_BEB7_Cattle_USA:0.00418910385098068,KF543867_CM5_Rhesus_macaque_China:0.00452041472999525)0.3090[stddev=1.4
4913767]:0.000372088791833791,KJ556988_HLJDV_Deer_China:0.00392749739393055)0.2380[stddev=1.33078924]:0.000486460967944768,PZ670833_CYG2_she
ep_China:0.00377736212262392)0.0860[stddev=0.85790442]:0.000654394043124637,PZ670836_BEB6_goat_China:-
0.000683722204927315)0.1300[stddev=1.06348484]:0.000346963506978034,PZ670828_BEB6_sheep_China:-
0.000346963506978034)0.0660[stddev=0.75099933]:0.00017611026490551,PX617835_BEB6_Cattle_Algeria:-
0.00017611026490551)0.2160[stddev=1.28802174]:0.000149610699752377,(PX617836_CM7_Cattle_Algeria:0.0,PZ670830_CM7_sheep_China:0.0)0.6190[stdd
ev=1.54240073]:0.00410634569113946)0.5150[stddev=1.58082257]:0.00531918055112751,PZ670831_CHG3_sheep_China:0.00335440086532415)0.5140[stddev
=1.58082257]:0.00216046409279241,(PZ670834_CHG5_goat_China:-
0.000573136106057467,(PZ670832_CHG1_sheep_China:0.0,PZ670837_CHG1_goat_China:0.0)0.5050[stddev=1.58113883]:0.00482372543680787)0.4570[stddev
=1.57321327]:0.00220431585394862)0.9990[stddev=0.31464265]:0.0349782913890033,KP262363_CHG10_Goat_China:0.01029416039483)0.6400[stddev=1.517
89328]:0.0246974910117106,((AY331007_BEB3_Cattle_USA:0.00331297690802603,PZ860264_BEB8_Cattle_Romania:0.00993847116383442)0.1800[stddev=1.21
490740]:0.00270615529154116,(KT984495_BEB17_Cattle_Brazil:0.00254280817884691,PZ846586_BEB17_Cattle_Romania:0.010882622742329)0.6120[stddev=
1.54240073]:0.00663424355007251)0.2030[stddev=1.26491106]:0.00206364800788644)0.4360[stddev=1.56556699]:0.0129766121185207,((((AY331008_BEB4
_Cattle_USA:-
0.000117451458820212,HM992510_CHN2_Human_China:0.00430688755169943)0.4380[stddev=1.56556699]:0.00361766358068393,PZ569993_BEB4_Cattle_Roman
ia:0.0331546375045133)0.1570[stddev=1.12915898]:0.00179363896580483,JQ044399_BEB9_Cattle_USA:0.00143703688897343)0.0990[stddev=0.90498619]:0
.00152314439051447,((((PX617833_I_Cattle_Algeria:0.0,PZ846585_I_Cattle_Romania:0.0)0.6740[stddev=1.48694317]:0.0,MH732748_l_cattle_China:0.0
)0.6450[stddev=1.51789328]:0.0,AF135836_I_Cattle_Germany:0.0)0.6450[stddev=1.51789328]:0.00390798439432406,(MN523335_SHR1_Rat_China:0.004234
87959479057,(AF135837_J_Cattle_Germany:-0.0000388139601032863,(KX383638_JLD-VIII_Sika_deer_China:-
0.0000517519468043819,(PX617832_J_Cattle_Algeria:-
0.0000776279202065727,PZ846587_J_Cattle_Romania:0.00876958386612905)0.2570[stddev=1.36930639]:0.0000517519468043819)0.1280[stddev=1.02761861
]:0.0000388139601032863)0.0880[stddev=0.85790442]:0.0000328799817294369)0.1230[stddev=1.02761861]:0.000332875559370875)0.2060[stddev=1.26491
106]:0.00126508652890899)0.2440[stddev=1.35055544]:0.00451058311762914)0.3770[stddev=1.52676128]:0.0058908625891502,(((((AY237212_WL4_Muskra
t_USA:0.000687451521764051,KF591686_WL25_Deer_mouse_USA:0.00359958499645236)0.5680[stddev=1.56971335]:0.0031616963710622,(AY237214_WL6_Muskr
at_USA:-
0.000983809439827198,DQ885584_PtEb_VIII_Cat_Portugal:0.00962176866147846)0.8460[stddev=1.15931014]:0.00568781371383632)0.9190[stddev=0.90498
619]:0.0183504130818948,KF591684_WL22_Woodchuck_USA:0.00969720916156865)0.3580[stddev=1.50831031]:0.00295461803936739,KF591685_VL23_Deer_mou
se_USA:0.0135477679504982)0.6400[stddev=1.51789328]:0.0192798948203932,(AF101197_A_Human:0.00081967149700168,((FJ439681_S5_Human_Malawi:-
0.000704325694376919,PX617837_Peru6_Cattle_Algeria:0.0136994142494092)0.5710[stddev=1.56556699]:0.00265244867214476,(PX617831_TypeIV_Cattle_
Algeria:-0.0000622023783721934,(PZ670829_TypeIV_sheep_China:-0.00012025793151957,(PZ670835_Type_goat_China:-
0.000232757286812075,(PV715457_Peru8_macaque_China:0.0000530771794695565,(AF101200_D_Human:-0.000161842911688685,(MK809249_D_peacock_China:-
0.000315167775393763,(LC436503_WL2_deer_Korea:0.00490931550658475,PX617834_D_Cattle_Algeria:-
0.000614173100767336)0.5120[stddev=1.58082257]:0.000315167775393763)0.6190[stddev=1.54240073]:0.000161842911688685)0.6250[stddev=1.53492671]
:0.00425696944751549)0.6450[stddev=1.51789328]:0.00452412207736593)0.3640[stddev=1.51789328]:0.00012025793151957)0.3600[stddev=1.51789328]:0
.0000622023783721934)0.5420[stddev=1.57607106]:0.00174210561848975)0.5510[stddev=1.57321327]:0.00347646056294752)0.7190[stddev=1.43492160]:0
.0154608739982484)0.0340[stddev=0.53944416]:0.00190022784424974)0.0890[stddev=0.85790442]:0.00549180997863089,((KJ867487_DeerEb8_white_taile
d_deer_USA:0.00942034329825963,KJ867488_DeerEb9_white_tailed_deer_USA:0.00360279021542886)0.4860[stddev=1.57987341]:0.00366387488032163,(KJ8
67484_DeerEb5_white_tailed_deer_USA:-
0.000198859845400452,(KJ867482_DeerEb3_white_tailed_deer_USA:0.0045256779184611,KJ867485_DeerEb6_white_tailed_deer_USA:-
0.000238641400244689)0.8950[stddev=0.98944429]:0.0089192416609699)0.5510[stddev=1.57321327]:0.00762565780744607)0.8220[stddev=1.21490740]:0.
0166260750285839)0.0130[stddev=0.31464265]:0.000731207022815407,(MN523336_SHR1_Rat_China:0.000643299949222117,(MK348513_MJ14_Binturong_China
:-
0.000498795112220634,MK909573_SCR06_Rabbit_China:0.00479393751803804)0.5350[stddev=1.57829021]:0.0036704805574257)0.5630[stddev=1.56971335]:
0.00454594971310341)0.0150[stddev=0.31464265]:0.00204503871408687,((((KR062121_WR10_Bank_vole_Poland:0.00458241692980358,KR062123_WR9_Yellow
_necked_mouse_Poland:0.0128748120628168)0.6840[stddev=1.47512711]:0.00887655085409455,KR062124_WR8_Striped_field_mouse_Poland:0.009048063130
78487)0.5490[stddev=1.57607106]:0.00578381630997867,KR062125_WR7_Striped_field_mouse_Poland:0.0391360955900467)0.8960[stddev=0.98944429]:0.0
228708583855177,((KT267293_CHB1_Black_Bear_China:-
0.000826889336082384,KX276711_SCH4_Horse_China:0.00509524727894313)1.0000[stddev=0.00000000]:0.0534174968384776,((JQ863269_WW1_Human_China:0
.00538394266942753,KU885972_HNWW4_Human_China:0.00327040280682477)0.5230[stddev=1.57987341]:0.00305257207985487,(JQ863270_WW2_Human_China:0.
000137908096766198,(JQ863271_WW3_Human_China:-
0.00176312664889472,(JQ863272_WW4_Human_China:0.0139443273266422,JQ863273_WW5_Human_China:0.0129231830382885)0.9790[stddev=0.53944416]:0.001
39481337412293)0.9840[stddev=0.44271887]:0.0178145489492359)0.4650[stddev=1.57607106]:0.00379132966885093)0.6270[stddev=1.53492671]:0.010506
5366385567)0.3060[stddev=1.44913767]:0.00377415144755329)0.1990[stddev=1.24056439]:0.00741113289228808)0.0370[stddev=0.53944416]:0.004864389
65614583,((FJ439683_S7_Human_Netherlands:-
0.00099626077634124,OR838688_NegP1_Guinea_Pig_China:0.00533876331353504)0.8980[stddev=0.98944429]:0.00981784460353479,(KY593707_EbRB_Human_B
razil:0.0034363218013591,PP905661_EBCMAP_019_Human_France:0.00973158535373151)0.5300[stddev=1.57829021]:0.00369608046692779)0.6040[stddev=1.
54919334]:0.00811306359959719)0.1490[stddev=1.09726934]:0.00277909075318647,(KT267287_CHK2_Gray_Kangaroo_China:0.00293870457517049,(KT267286
_CHK1_White_Kangaroo_China:0.0,KY706127_CSK1_White_Kangaroo_China:0.0)0.6630[stddev=1.49799866]:0.00132965336769026)1.0000[stddev=0.00000000
]:0.0696062031935387)0.8350[stddev=1.18785521]:0.0391978863278257,((((((MH940283_RS01_Squirrel_China:-
0.00392151094719809,MH940284_RS02_Squirrel_China:0.0126134668931206)0.7690[stddev=1.35055544]:0.00809729260620332,MF410401_SCC2_Chipmunk_Chi
na:-
0.00373222908575873)0.3470[stddev=1.49799866]:0.00340039724750956,(ON165751_XJHT4_Marmota_China:0.0125862009556923,(MF410402_SSCC3_Chipmunk_
China:-
0.000190193764465587,MF410403_SCC4_Chipmunk_China:0.00445892889321857)0.3000[stddev=1.44913767]:0.000772506570118623)0.1960[stddev=1.2405643
9]:0.00114135688776903)0.3400[stddev=1.49799866]:0.00189931148807799,OR838690_NESQ1_Squirrel_China:0.000278260594573216)0.6090[stddev=1.5491
9334]:0.00678738951273717,MF410400_SCC1_Chipmunk_China:0.00419424345913442)0.8460[stddev=1.15931014]:0.0495194517572758,((((KF543866_CM4_Mon
key_China:-
0.00223115305472581,KJ668732_CD5_Dog_China:0.00653626409080305)0.3710[stddev=1.52676128]:0.00326233683696099,KJ728811_CM18_Monkey_China:0.00
104760551493424)0.4540[stddev=1.57321327]:0.00232916881937083,KM591951_XH3_Monkey_China:0.00636165545957776)0.9500[stddev=0.68920244]:0.0774
291248424531,((((DQ683749_CAF4_Human_Gabon:0.0138204880551802,DQ885588_PtEb_XII_Marmoset_Portugal:-
0.0138204880551802)0.7600[stddev=1.35055544]:0.0238544614215112,JQ437575_KIN3_Human_Congo:-
0.00601127789531764)0.4890[stddev=1.57987341]:0.0110133908456856,JF681180_KB6_Baboon_Kenya:-
0.00232085264095961)1.0000[stddev=0.00000000]:0.351239124489516,(JQ863275_WW7_Human_China:0.00263344400284624,((AY237210_WL2_Raccoon_USA:-
0.0053601990438096,AY237211_WL3_Raccoon_USA:0.00966531007988679)0.4160[stddev=1.55531347]:0.00302976883585001,(AY237209_WL1_Raccoon_USA:0.00
0465056358202362,(JQ863274_WW6_Human_China:0.00871716574045309,KF591687_WL26_Racoon_USA:-
0.00441205470437581)0.7630[stddev=1.35055544]:0.0176689755698452)0.3550[stddev=1.50831031]:0.00635102169174466)0.7120[stddev=1.43492160]:0.0
273151147596398)0.9940[stddev=0.31464265]:0.222255492448665)0.9980[stddev=0.31464265]:0.229296691938467)0.6350[stddev=1.52676128]:0.03231044
11035724)0.9560[stddev=0.68920244]:0.095955974007374)0.7410[stddev=1.38708327]:0.0296581197149243,(JN997480_25005-
Nig4_Human:0.0169658663442714,(JX524494_Nig7_Human_Nigeria:0.00549503039701252,JX683806_31372_Human_Nigeria:-
0.00118991936093527)0.2250[stddev=1.30996183]:0.000906012719293885)0.4240[stddev=1.56076904]:0.0456661338802812)0.3010[stddev=1.44913767]:0.
0325621998266229,(JN595887_17242259_Human:0.000574668320781849,JQ837797_gorilla3_Gorilla_Central_Africa:0.00817766309584882)0.9990[stddev=0.
31464265]:0.0594324790482095)0.2240[stddev=1.30996183]:0.028086338526638,KU194606_XJH4_Horse_China:0.0117485786352394)0.2680[stddev=1.387083
27]:0.0139236962892816,(KX276712_YNH1_Horse_China:0.000882932037795771,(JF681179_KB5_Baboon_Kenya:-
0.00163709592500233,(GQ406054_horse_2_Horse_Colombia:-
0.00127431133743693,(KU194603_XJH1_Horse_China:0.00394079155292159,(KU847350_CE01_Squirrel_China:0.00460823934991357,KX276714_YNH2_Horse_Chi
na:0.00406622554884849)0.4710[stddev=1.57829021]:0.000396440896459448)0.6630[stddev=1.49799866]:0.00124218992413316)0.3700[stddev=1.52676128
]:0.00594703827689755)0.3850[stddev=1.53492671]:0.00344969262177278)0.6200[stddev=1.53492671]:0.0204273967361885)0.4640[stddev=1.57607106]:0
.0566228612711711,JX000572_Macaque1_Macaque_China:-0.00886297374959815,(JQ863277_19079_Wastewater:-
0.0014072383659873,((JQ863276_WW8_Human_China:-
0.00121476918800933,KJ668735_CD8_Dog_China:0.00561459285736974)0.4110[stddev=1.55531347]:0.00364365455223006,(DQ885585_PtEb_IX_Dog_Australia
:-0.0074780454488319,(KJ668734_CD7_Dog_China:-0.00498631906180753,(KM870517_NED3_Dog_China:-
0.00966735501929846,KM870518_NED4_Dog_China:0.0185533944100119)0.3950[stddev=1.54240073]:0.00938172026880355)0.5370[stddev=1.57829021]:0.007
45161937270186)0.3420[stddev=1.49799866]:0.0124369385151339)0.3810[stddev=1.53492671]:0.00706455709683421)1.0000[stddev=0.00]:1.048950197979
39);

Table S1. *E. bieneusi* ITS sequences included in the phylogenetic analyses

GenBank accession number, genotype, phylogenetic group, host, and country of origin are provided for each sequence. The final column indicates inclusion in the restricted phylogenetic analysis comprising Groups 1, 2, 3, 8, and 14. A total of 117 sequences were included in the complete analysis, of which 56 were selected for the restricted analysis.

GenBank accession no.	Genotype	Phylogenetic group	Host	Country of origin	Restricted analysis
AF101197	A	Group1	Human	Germany	YES
AF101200	D	Group1	Human	Germany	YES
AF135836	I	Group2	Cattle	Germany	YES
AF135837	J	Group2	Cattle	Germany	YES
AY237209	WL1	Group4	Raccoon	USA	
AY237210	WL2	Group4	Raccoon	USA	
AY237211	WL3	Group4	Raccoon	USA	
AY237212	WL4	Group3	Muskrat	USA	YES
AY237214	WL6	Group3	Muskrat	USA	YES
AY331007	BEB3	Group2	Cattle	USA	YES
AY331008	BEB4	Group2	Cattle	USA	YES
DQ683749	CAF4	Group5	Human	Gabon	
DQ885584	PtEb VIII	Group3	Cat	Portugal	YES
DQ885585	PtEb IX	Group11	Dog	Australia	
DQ885588	PtEb XII	Group5	Marmoset	Portugal	
EU153585	BEB7	Group2	Cattle	USA	YES
FJ439681	S5	Group1	Human	Malawi	YES
FJ439683	S7	Group10	Human	Netherlands	
GQ406054	horse 2	Group6	Horse	Colombia	
HM992510	CHN2	Group2	Human	China	YES
JF681179	KB5	Group6	Baboon	Kenya	
JF681180	KB6	Group5	Baboon	Kenya	
JN595887	MAY1	Group6	Human	France	
JN997480	Nig4	Group12	Human	Nigeria	
JQ044399	BEB9	Group2	Cattle	USA	YES
JQ437575	KIN3	Group5	Human	Congo	
JQ837797	gorilla3	Group6	Gorilla	Central Africa	
JQ863269	WW1	Group9	Human	China	
JQ863270	WW2	Group9	Human	China	
JQ863271	WW3	Group9	Human	China	
JQ863272	WW4	Group9	Human	China	
JQ863273	WW5	Group9	Human	China	
JQ863274	WW6	Group9	Human	China	
JQ863275	WW7	Group9	Human	China	
JQ863276	WW8	Group9	Human	China	
JQ863277	WW9	Group9	Human	China	
JX000572	Macaque1	Group6	Macaque	China	
JX524494	Nig7	Group12	Human	Nigeria	
JX683806	Nig6	Group12	Human	Nigeria	
KF543866	CM4	Group7	Monkey	China	
KF543867	CM5	Group2	Rhesus macaque	China	YES
KF591684	WL22	Group3	Woodchuck	USA	YES
KF591685	VL23	Group3	Deer mouse	USA	YES
KF591686	WL25	Group3	Deer mouse	USA	YES
KF591687	WL26	Group4	Racoon	USA	
KJ556988	HLJDV	Group2	Deer	China	YES
KJ668732	CD5	Group7	Dog	China	
KJ668734	CD7	Group11	Dog	China	
KJ668735	CD8	Group11	Dog	China	
KJ728811	CM18	Group7	Monkey	China	
KJ867482	DeerEb3	Group8	White tailed deer	USA	YES
KJ867484	DeerEb5	Group8	White tailed deer	USA	YES
KJ867485	DeerEb6	Group8	White tailed deer	USA	YES
KJ867487	DeerEb8	Group8	White tailed deer	USA	YES
KJ867488	DeerEb9	Group8	White tailed deer	USA	YES
KM591951	XH3	Group7	Monkey	China	
KM870517	NED3	Group11	Dog	China	
KM870518	NED4	Group11	Dog	China	
KP262363	CHG10	Group2	Goat	China	YES
KR062121	WR10	Group9	Bank vole	Poland	
KR062123	WR9	Group9	Yellow necked mouse	Poland	
KR062124	WR8	Group9	Striped field mouse	Poland	
KR062125	WR7	Group9	Striped field mouse	Poland	
KT267286	CHK1	Group13	White kangaroo	China	
KT267287	CHK2	Group13	Gray kangaroo	China	
KT267293	CHB1	Group13	Black bear	China	
KT984495	BEB17	Group2	Cattle	Brazil	YES

GenBank accession no.	Genotype	Phylogenetic group	Host	Country of origin	Restricted analysis
KU194603	XJH1	Group6	Horse	China	
KU194606	XJH4	Group6	Horse	China	
KU847350	SCYA	Group6	Squirrel	China	
KU885972	HNWW4	Group9	Human	China	
KX276711	SCH4	Group13	Horse	China	
KX276712	YNH1	Group6	Horse	China	
KX276714	YNH2	Group6	Horse	China	
KX383638	JLD-VIII	Group2	Sika deer	China	YES
KY593707	EbRB	Group10	Human	Brazil	
KY706127	CSK1	Group13	White kangaroo	China	
LC436503	WL2	Group1	Deer	Korea	YES
MF410400	SCC1	Group15	Chipmunk	China	
MF410401	SCC2	Group15	Chipmunk	China	
MF410402	SCC3	Group15	Chipmunk	China	
MF410403	SCC4	Group15	Chipmunk	China	
MH732748	I	Group2	Cattle	China	YES
MH940283	SCRS03	Group15	Squirrel	China	
MH940284	SCRL04	Group15	Squirrel	China	
MK348513	MJ14	Group14	Binturong	China	YES
MK809249	D	Group1	Peacock	China	YES
MK909573	SCR06	Group14	Rabbit	China	YES
MN523335	SHR1	Group2	Rat	China	YES
MN523336	SHR1	Group14	Rat	China	YES
ON165751	XJHT4	Group15	Marmota	China	
OR838688	NegP1	Group10	Guinea pig	China	
OR838690	NESQ1	Group15	Squirrel	China	
PP905661	EBCMAP	Group10	Human	France	
PV715457	Peru8	Group1	Macaque	China	YES
PX617831	TypeIV	Group1	Cattle	Algeria	YES
PX617832	J	Group2	Cattle	Algeria	YES
PX617833	I	Group2	Cattle	Algeria	YES
PX617834	D	Group1	Cattle	Algeria	YES
PX617835	BEB6	Group2	Cattle	Algeria	YES
PX617836	CM7	Group2	Cattle	Algeria	YES
PX617837	Peru6	Group1	Cattle	Algeria	YES
PZ670828	BEB6	Group2	Sheep	China	YES
PZ670829	Type	Group1	Sheep	China	YES
PZ670830	CM7	Group2	Sheep	China	YES
PZ670831	CHG3	Group2	Sheep	China	YES
PZ670832	CHG1	Group2	Sheep	China	YES
PZ670833	CYG2	Group2	Sheep	China	YES
PZ670834	CHG5	Group2	Goat	China	YES
PZ670835	TypeIV	Group1	Goat	China	YES
PZ670836	BEB6	Group2	Goat	China	YES
PZ670837	CHG1	Group2	Goat	China	YES
PZ846585	I	Group2	Cattle	Romania	YES
PZ846586	BEB17	Group2	Cattle	Romania	YES
PZ846587	J	Group2	Cattle	Romania	YES
PZ860264	BEB8	Group2	Cattle	Romania	YES
PZ569993	BEB4	Group2	Cattle	Romania	YES

Supplementary Dataset S2. Multiple – sequence alignment of the restricted ITS dataset (56 sequences; Groups 1, 2, 3, 8, and 14)

Multiple-sequence alignment used for the restricted phylogenetic analysis. The dataset comprises 56 selected ITS sequences representing Groups 1, 2, 3, 8, and 14, including all five Romanian cattle-derived sequences.

>AF101197_A_Human
ATTTTCAGTTTTTGGGGTGTGGGTATCGGAATGTGTGGTAGGTGATGTGTGTGTGTATGGGGGATGCCGAGGGGACCAGCGGTGCGGTGGTGTGTGTAGGCGTGAGAGTGTATCTGCAAGGGTGAGGGA
TGTGGGTGCAGTGAGTTAGAGGTGGTTCCATGTGGAATAGTGGGATTGGTACGTGATGGTTGGATGGGGGAATGATGTGTGTATGGGTGAGGAAAATCGGAGGTTGC
>AF101200_D_Human
ATTTTCAGTTTTTGGGGTGTGGGTATCGGAATGTGTGGTAGGTGATGTGTGTGTGTATGGGGGATGCCGAGGGGACCAGCGGTGCGGTGGTGTGTGCAGGCGTGAGAGTGTATCTGCAAGTGTGAGGGA
TGTGGGTGCAGCGAGTTAGAGGTGGTTCCATGTGGAATAGTGGGATTGGTACGTGATGGTTGGATGGGGGAATGATGTGTGTATGGGTGAGGAAAATCGGAGGTTGC
>AF135836_I_Cattle_Germany
ATTTTCAGTTTTTGGGGTGTGGGTATCGGAATGTGTGATAGGTGATGTGTGTGTGTATGGGGGATGCCGAGGGGACCAGCGGTGTGGTGGTGTGTGTAGGCGTGAGAGTGTATCTGTAAGGGTGAGGGA
TGTAGCTGCAGTGAGTTAGAGATGGTTCCATGAGGAATAGTGGGATTGGTACGTGATGGTTGGATGGGGGAATGATGTGTGTATGGGTGAGGAAAATCGGAGGTTGC
>AF135837_J_Cattle_Germany
ATTTTCAGTTTTTGGGGTGTGGGTATCGGAATGTGTGGTAGGTGATGTGTGTGTGTATGGGGGATGCCGAGGGGACCAGCGGTGTGGTGGTGTGTGTAGGCGTGAGAGTGTATCTGTAAGGGTGAGGGA
TGTAGCTGCAGTGAGTTAGAGATGGTTCCATGAGGAATAGTGGGATTGGTACGTGATGGTTGGATGGGGGAATGATGTGTGTATGGGTGAGGAAAATCGGAGGTTGC
>AY237212_WL4_Muskrat_US
ATTTTCAGTTTTTAGGGTGTGGGTATCGGAATGTATTGTGAGTGA--
TGTGTGTGTATAGGGGATGCCGAGGGGACCAGCGGTGTGGTGGTATGTGTAGATGTGAGAGTGTATCTGTAAGGGTGAGGGATGTGGCTGCAGTGAGTTAGAGGTGGTTCCATGAGGAATAGTGGGATT
GGTACGTGATGGTTGGATGGGAGAATGATGTGTGTATAGGTGAGGAAAATCGGAGGTTGC
>AY237214_5540_Muskrat
ATTTTCAGTTTTTAGGGTGTGGGTATCGGAATGTATTGTGAGTGA--
TGTGTGTGTATAGAGGATGCCGAGGGGACCAGCGGTGTGGTGGTATGTGTAGATGTGAGAGTGTATCTGTAAGGGTGAGGGATGTGGCTGCAGTGAGTTAGAGGTGGTTCCATGAGGAATAGTGGGATT
GGTACGTGATGGTTGGATGGGGGAATGATGTGTGTATAGGTGAGGAAAATCGGAGGTTGC
>AY331007_BEB3_Cattle_USA
ATTTTCAGTTTTTAGGGTGTGAGTATCGGAATGTATAGTAGGTGATGTGTGTGTGTATGGGGGATGCCGAGGGGACCAGCGGTGCGGTGGTGTGTGTAGGCGTGAGAGCGTATCTGTAAGTGTGAGGGA
TGTAGCTGCAGTGAGTTAGAGATGGTTCCATGAGGAATAGTGGGATTGGTACGTGATGGTTGGATGGGGGAATGATGTGTGTATGGGTGAGGAAAATCGGAGGTTGC
>AY331008_BEB4_Cattle_USA
ATTTTCAGTTTTTGGGGTGTGAGTATCGGAATGTGTAGTAGGTGATGTGTGTGTGTATGGGGGATGCCGAGGGGACCAGCGGTGTGGTGGTGTGTGTAGGCGTGAGAGTGTATCTGTAAGGGTGAGGGA
TGTAGCTGCAGTGAGTTAGAGATGGTTCCATGAGGAATAGTGGGATTGGTACGTGATGGTTGGATGGGGGAATGATGTGTGTATGGGTGAGGAAAATCGGAGGTTGC
>DQ885584_PtEb_VIII_Cattle_Portugal
ATTTTCAGTTTTTAGGGTGTGGGTATCGGAATGTATTGTGAGTGA--
TGTGTGTGTATAGAGGATGCCGAGGGGACCAGCGGTGTGGTGGTACGTGTAGATGTGAGAGTGTATCTGTAAGGGTGAGGGATGTGGCTGCAGTGAGTTAGAGGTGGTTCCATGAGGAATAGTGGGGTT
GGTACGTGATGGTTGGATGGGGGAATGATGTGTGTATAGGTGAGGAAAATCGGAGGTTGC
>EU153585_BEB7_Cattle_USA
ATTTTCAGTTTTTAGGGTGTGAGTATCGGAATGTATAGTAGGTGATGTATGTGTGTATGGGTGATGCCGAGGGGACCCGCTGTGCGGTGGTGTGTGTAGGCGTGAGAGTGTATCTGTAAGTGTGAGGGA
TGTAGCTGCAGTGAGTTAGAGATGGTTCCATGAGGAATAGTGGGATTGGTACACGATGGGTTGGATGGAGAATGATGTGTGTATGGGTGAGGAAAATCGGAGGTTGC
>FJ439681_S5_Human_Malawi
ATTTTCAGTTTTTGGGGTGTGGGTATCGGAATGTATGGTAGGTGATGTGTGTGTGTATGGGGGATGCCGAGGGGACCAGCGGTGCGGTGGTGTGTGTAGGCGTGAGAGTGTATCTGCAAGGGTGAGGGA
TGTGGGTGCAGCGAGTTAGAGGTGGTTCCATGTGGAATAGTGGGATTGGTACGTGATGGTTGGATGGGGGAATGATGTGTGTATGGGTGAGGAAAATCGGAGGTTGC
>HM992510_CHN2_Human_China
ATTTTCAGTTTTTGTGGTGTGAGTATCGGAATGTGTAGTAGGTGATGTGTGTGTGTATGGGGGATGCCGAGGGGACCAGCGGTGTGGTGGTGTGTGTAGGCGTGAGAGTGTATCTGTAAGGGTGAGGGA
TGTAGCTGCAGTGAGTTAGAGATGGTTCCATGAGGAATAGTGGGATTGGTACGTGATGGTTGGATGGGGGAATGATGTGTGTATGGGTGAGGAAAATCGGAGGTTGC
>JQ044399_BEB9_Cattle_USA
ATTTTCAGTTTTTGGGGTGTGGGTATCGGAATGTGTAGTAGGTGATGTGTGTGTGTATGGGGGATGCCGAGGGGACCAGCGGTGTGGTGGTGTGTGTAGGCGTGAGAGTGTATCTGTAAGGGTGAGGGA
TGTAGCTGCAGTGAGTTAGAGATGGTTCCATGAGGAATAGTGGGATTGGTACGTGATGGTTGGATGGGGGAATGATGTGTGTATGGGTGAGGAAAATCGGAGGTTGC
>KF543867_CM5_rhesus_macaque
ATTTTCAGTTTTTAGGGTGTGAGTATCGGAATGTATAGTAGGTGATGTATGTGTGTATGGGTGATGCCGAGGGGACCCGCGGTGCGGTGGTGTGTGTAGGCGTGATAGTGTATCTGTAAGTGTGAGGGA
TGTAGCTGCAGTGAGTTAGAGATGGTTCCATGAGGAATAGTGGGATTGGTACACGATGGGTTGGATGGAGAATGATGTGTGTATGGGTGAGGAAAATCGGAGGTTGC
>KF591684_13484_Woodchuck
ATTTTCAGTTTTTAGGGTGTGGGTATCGGAATGTGTGGTAGGTGATGTGTGTGTGTATGGGGGATGCCGAGGGGACCAGCGGTGTGGTGGTATGTGTAGATGTGAGAGTGTATCTGTAAGGGTGAGGGA
TGTGGCTGCAGTGTGTTAGAGGTGGTTCCATGAGGAATAGTGGGATTGGTACGTGNTGGTTGGATGGGAGAATGATGTGTGTATAGGTGAGGAAAATCGGAGGTTGC
>KF591685_VL23_Deer_mouse_USA
ATTTTCAGTTTTTAGGGTGTGGGTATCGGAATGTGTGGTAGGTGATGTGTGTGTGTATGGGGGATGCCGAGGGGACCAGCGGTGTGGTGGTATGTGCAGACGTGAGAGTGTATCTGCAAGGGTGAGGGA
TGTGGCTGCAGCGAGTTAGAGGTGGTTCCATGAGGAATAGTGGGATTGGTACGTGATGGTTGGATGGGAGAATGATGTGTGTATAGGTGAGGAAAATCGGAGGTTGC
>KF591686_12977_Deer_mouse
ATTTTCAGTTTTTAGGGTGTGGGTATCGGAATGTATTGTGAGTGA--
TGTGTGTGTATAGGGGATGCCGAGGGGACCAGCGGTGTGGTGGTATGTGTAGATGTGAGAGTGTATCTGTAAGGGTGAGGGATGTGGCTGCAGCGAGTTAGAGGTGGTTCCATGAGGAATAGTGGGATT
GGTACGTGATGGTTGGATGGGAGAATGATGTGTGTATAGGTGAGGAAAATCGGAGGTTGC
>KJ556988_HLJDV_Deer_China
ATTTTCAGTTTTTAGGGTGTGAGTATCGGAATGTATAGTAGGTGATGTATGTGTGTATGGGTGATGCCGAGGGGACCCGCGGTGCGGTGGTGTGTGTAGGCGTGAGAGTGTATCTGTAAGTGTGAGGGA
TGTAGCTGCAGTGAGTTAAAGATGGTTCCATGAGGAATAGTGGGATTGGTACACGATGGGTTGGATGGAGAATGATGTGTGTATGGGTGAGGAAAATCGGAGGTTGC
>KJ867482_DeerEb3_white_tailed_deer_USA
ATTTTCAGTTTTTAGGGTGTGTGTATCGGAGTGTATGACAGGTGA--
TGTGTGTGTATGGGGGATGCTGAGGGGACCAGTGGTGTGGTGGTGTGTGTAGGCGTGAGAGTGTATCTGTAAGGGTGAGGGATGTGGCTGCAGTGAGTTAGAGGTGGTTCCATGGGGAATAGTGGGATT
GGTACGTGATAGTTGGATAGGGGAATGATGTGTGTATGGGTGAGGAAAATCGGAGGTTGC
>KJ867484_DeerEb5_white_tailed_deer_USA
ATTTTCAGTTTTTAGGGTGTGTGTATCGGAATGTATGGTAGGTGATGTGTGTGTGTATGGGGGATGCTGAGGGGACCAGTGGTGTGGTGGTGTGTGTAGGCGTGAGAGTGTATCTGTAAGGGTGAGGGA
TGTGGCTGCAGTGAGTTAGAGGTGGTTCCATGGGGAATAGTGGGATTGGTACGTGATAGTTGGATAGGGGAATGATGTGTGTATGGGTGAGGAAAATCGGAGGTTGC
>KJ867485_DeerEb6_white_tailed_deer_USA
ATTTTCAGTTTTTAGGGTGTGTGTATCGGAGTGTATGATAGGTGA--
TGTGTGTGTATGGGGGATGCTGAGGGGACCAGTGGTGTGGTGGTGTGTGTAGGCGTGAGAGTGTATCTGTAAGGGTGAGGGATGTGGCTGCAGTGAGTTAGAGGTGGTTCCATGGGGAATAGTGGGATT
GGTACGTGATAGTTGGATAGGGGAATGATGTGTGTATGGGTGAGGAAAATCGGAGGTTGC
>KJ867487_DeerEb8_white_tailed_deer_USA
ATTTTCAGTTTTTAGGGTGTGTGTATCGGAGTGTATGATAGGTGATGTGTGTGTGTATGGGGGATGCTGAGGGGACCAGTGGTGTGGTGGTGTGTGTAGGCGTGAGAGTGTATCTGTAAGGGTGAGGGA
TGTAGCTGCAGTGAGTTAGAGGTGGTTCCATGGGGAATAGTGGGATTGGTACGTGATGGTTGGATGGGGGAATGATGTGTGGATGGGTGAGGAAAATCGGAGGTTGC
>KJ867488_DeerEb9_white_tailed_deer_USA
ATTTTCAGTTTTTAGGGTGTGTGTATCGGAATGTATGATAGGTGATGCGTGTGTGTATGGGGGATGCTGAGGGGACCAGTGGTGTGGTGGTGTGTGTAGGCGTGAGAGTGTATCTGTAAGGGTGAGGGA
TGTAGCTGCAGTGAGTTAGAGGTGGTTCCATGGGGAATAGTGGGATTGGTACGTGATGGTTGGATGGGGGAATGATGTGTGTATGGGTGAGGAAAATCGGAGGTTGC
>KP262363_CHG10_Goat_China
ATTTTCAGTTTTTGGGGTGTGGGTATCGGAATGTATGGTAGGTGATGTATGTGTGTATGGGGGATGCCGAGGGGACCCGCGGTGCGGTGGTGTGTGTAGGCGTGAGAGTGTATCTGTAAGGGTGAGGGA
TGTGGGTGCAGCGAGTTAGAGATGGTTCCATGAGGAATAGTGGGATTGGTACACGATGGGTGGAAGGGAGAATGATGTGTGTATGGGTGAGGAAAATCGGAGGTTGC
>KT984495_BEB17_Cattle_Brazil
ATTTTCAGTTTTTGGGGTGTGAGTATCGGAATGTGTAGTAGGTGATGTGTGTGTGTATGGGGGATGCCGAGGGGACCCGCGGTGCGGTGGTGTGTGTAGGCGTGAGAGCGTATCTGTAAGTGTGAGGGA
TGTAGCTGCAGTGAGTTAGAGATGGTTCCATGAGGAATAGTGGGATTGGTACGTGATGGTTGGATGGGGGAATGATGTGTGTATGGGTGAGGAAAATCGGAGGTTGC
>KX383638_JLD-VIII_Sika_deer_China
ATTTTCAGTTTTTGGGGTGTGGGTATCGGAATGTGTGGTAGGTGATGTGTGTGTGTATGGGGGATGCCGAGGGGACCAGCGGTGTGGTGGTGTGTGTAGGCGTGAGAGTGTATCTGTAAGGGTGAGGGA
TGTAGCTGCAGTGAGTTAGAGATGGTTCCATGAGGAATAGTGGGATTGGTACGTGATGGTTGGATGGGGGAATGATGTGTGTATGGGTGAGGAAAATCGGAGGTTGC
>LC436503_WL2_deer_Korea
ATTTTCAGTTTTTGGGGTGTGGGTATCGGAATGTGTGGTAGGTGATGTGTGTGTGTATGGGGGATGCCGAGGGGACCAGCGGTGCGGTGGTGTGTGCAGGCGTGAGAGTGTATCTGCAAGTGTGAGGGA
TGTGGGTGCAGCGAGTTAGAGGTGGTTCCATGTGGAATAGTGTGATTGGTACGTGATGGTTGGATGGGGGAATGATGTGTGTATGGGTGAGGAAAATCGGAGGTTGC
>MH732748_l_cattle
ATTTTCAGTTTTTGGGGTGTGGGTATCGGAATGTGTGATAGGTGATGTGTGTGTGTATGGGGGATGCCGAGGGGACCAGCGGTGTGGTGGTGTGTGTAGGCGTGAGAGTGTATCTGTAAGGGTGAGGGA
TGTAGCTGCAGTGAGTTAGAGATGGTTCCATGAGGAATAGTGGGATTGGTACGTGATGGTTGGATGGGGGAATGATGTGTGTATGGGTGAGGAAAATCGGAGGTTGC
>MK348513_MJ14_Binturong_China
ATTTTCAGTTTTTAGGGTGTGGGTATCGGAATGCATGGTAGGTGATGTGTGTGTGTATGGGGGATGCCGAGGGGACCAGCGGTGTGGTGGTGTGTGTAGGCGTGAGAGTGTATCTGTAAGGGTGAGGGA
TGTGGCTGCAGTGAGTTAGAGGTGGTTCCATGGGGAATAGTGGGATTGGTACGTGATGGTTGGATGGGGGAATGATGTGTGTATGGGTGAGGAAAATCGGAGGTTGC

```
>MK809249_D_peacock
ATTTTCAGTTTTTGGGGTGTGGGTATCGGAATGTGTGGTAGGTGATGTGTGTGTGTATGGGGGATGCCGAGGGGACCAGCGGTGCGGTGGTGTGTGCAGGCGTGAGAGTGTATCTGCAAGTGTGAGGGA
TGTGGGTGCAGCGAGTTAGAGGTGGTTCCATGTGGAATAGTGGGATTGGTACGTGATGGTTGGATGGGGGAATGATGTGTGTATGGGTGAGGAAAATCGGAGGTTGC
>MK909573_SCR06_Rabbit_China
ATTTTCAGTTTTTAGGGTGTGGGTATCGGAATGCATGGTAGGTGATGTGTGTGTGTATGGGGGATGCCGAGGGGACCAGCGGTGTGGTGGTGTGTGTAGGCGTGAGAGTGTATCTGTAAGGGTGAGGGA
TGTGGCTGCAGTGAGTTAGAGGTGGTTCCATGGGGAATAGTGGGATTGGTACGTGATGGTTGGATGGGGGAATGATGTGTGTATGGGTGAGGAAACTCGGAGGTTGC
>MN523335_SHR1_Rat_China
ATTTTCAGTTTTTGGGGTGTGGGTATCGGAATGTGTGGTAGGTGATGTGTGTGTGTATGGGGGATGCCGAGGGGACCAGCGGTGTGGTGGTGTGTGTAGGTGTGAGAGTGTATCTGTAAGGGTGAGGGA
TGTAGCTGCAGTGAGTTAGAGATGGTTCCATGAGGAATAGTGGGATTGGTACGTGATGGTTGGATGGGGGAATGATGTGTGTATGGGTGAGGAAAATCGGAGGTTGC
>MN523336_156_Rat
ATTTTCAGTTTTTAGGGTGTGGGTATCGGAATGCATGGTAGGTGATGTGTGTGTGTATGGGGGATGCCGAGGGGACCAGCGGTGTGGTGGTGTGTGTAGGCGTGAGAGTGTATCTGTAAGGGTGAGGGA
TGTGGCTGCAGTGAGTTAGAGATGGTTCCATGGGGAATAGTGGGATTGGTACGTGATGGTTGGATGGGGGAATGATGTGTGTATGGGTGAGGAAAATCGGAGGTTGC
>PV715457_Peru8_macaque
ATTTTCAGTTTTTGGGGTGTGGGTATCGGAATGTGTGGTAGGTGATGTGTGTGTGTATGGGGGATGCCGAGGGGACCAGCGGTGCGGTGGTGTGTGCAGGCGTGAGAGTGTATCTGCAAGGGTGAGGGA
TGTGGGTGCAGCGAGTTAGAGGTGGTTCCATGTGGAATAGTGGGATTGGTACGTGATGGTTGGATGGGGGAATGATGTGTGTATGGGTGAGGAAAATCGGAGGTTGC
>PX617831_Cattle_Algeria
ATTTTCAGTTTTTGGGGTGTGGGTATCGGAATGTGTGGTAGGTGATGTGTGTGTGTATGGGGGATGCCGAGGGGACCAGCGGTGCGGTGGTGTGTGTAGGCGTGAGAGTGTATCTGCAAGGGTGAGGGA
TGTGGGTGCAGCGAGTTAGAGGTGGTTCCATGTGGAATAGTGGGATTGGTACGTGATGGTTGGATGGGGGAATGATGTGTGTATGGGTGAGGAAAATCGGAGGTTGC
>PX617832_Cattle_Algeria
ATTTTCAGTTTTTGGGGTGTGGGTATCGGAATGTGTGGTAGGTGATGTGTGTGTGTATGGGGGATGCCGAGGGGACCAGCGGTGTGGTGGTGTGTGTAGGCGTGAGAGTGTATCTGTAAGGGTGAGGGA
TGTAGCTGCAGTGAGTTAGAGATGGTTCCATGAGGAATAGTGGGATTGGTACGTGATGGTTGGATGGGGGAATGATGTGTGTATGGGTGAGGAAAATCGGAGGTTGC
>PX617833_Cattle_Algeria
ATTTTCAGTTTTTGGGGTGTGGGTATCGGAATGTGTGATAGGTGATGTGTGTGTGTATGGGGGATGCCGAGGGGACCAGCGGTGTGGTGGTGTGTGTAGGCGTGAGAGTGTATCTGTAAGGGTGAGGGA
TGTAGCTGCAGTGAGTTAGAGATGGTTCCATGAGGAATAGTGGGATTGGTACGTGATGGTTGGATGGGGGAATGATGTGTGTATGGGTGAGGAAAATCGGAGGTTGC
>PX617834_Cattle_Algeria
ATTTTCAGTTTTTGGGGTGTGGGTATCGGAATGTGTGGTAGGTGATGTGTGTGTGTATGGGGGATGCCGAGGGGACCAGCGGTGCGGTGGTGTGTGCAGGCGTGAGAGTGTATCTGCAAGTGTGAGGGA
TGTGGGTGCAGCGAGTTAGAGGTGGTTCCATGTGGAATAGTGGGATTGGTACGTGATGGTTGGATGGGGGAATGATGTGTGTATGGGTGAGGAAAATCGGAGGTTGC
>PX617835_Cattle_Algeria
ATTTTCAGTTTTTAGGGTGTGAGTATCGGAATGTATAGTAGGTGATGTATGTGTGTATGGGTGATGCCGAGGGGACCCGCGGTGCGGTGGTGTGTGTAGGCGTGAGAGTGTATCTGTAAGTGTGAGGGA
TGTAGCTGCAGTGAGTTAGAGATGGTTCCATGAGGAATAGTGGGATTGGTACACGATGGGTTGGATGGAGAATGATGTGTGTATGGGTGAGGAAAATCGGAGGTTGC
>PX617836_Cattle_Algeria
ATTTTCAGTTTTTAGGGTGTGAGTATCGGAATGTATAGTAGGTGATGTATGTGTGTATGGGTGATGCCGAGGGGACCCGTGGTGCGGTGGTGTGTGTAGGCGTGAGAGTGTATCTGTAAGTGTGAGGGA
TGTAGCTGCAGTGAGTTAGAGATGGTTCCATGAGGAATAGTGGGATTGGTACACGATGGGTTGGATGGAGAATGATGTGTGTATGGGTGAGGAAAATCGGAGGTTGC
>PX617837_Cattle_Algeria
ATTTTCAGTTTTTGGGGTGTGGGTATCGGAATGTATGGTAGATGATGTGTGTGTGTATGGGAGATGCCGAGGGGACCAGCGGTGCGGTGGTGTGTGTAGGCGTAAGAGTGTATCTGCAAGGGTGAGGGA
TGTGGGTGCAGCGAGTTAGAGGTGGTTCCATGTGGAATAGTGGGATTGGTACGTGATGGTTGGATGGGGGAATGATGTGTGTATGGGTGAGGAAAATCGGAGGTTGC
>PZ670828_BEB6_sheep
ATTTTCAGTTTTTAGGGTGTGAGTATCGGAATGTATAGTAGGTGATGTATGTGTGTATGGGTGATGCCGAGGGGACCCGCGGTGCGGTGGTGTGTGTAGGCGTGAGAGTGTATCTGTAAGTGTGAGGGA
TGTAGCTGCAGTGAGTTAGAGATGGTTCCATGAGGAATAGTGGGATTGGTACACGATGGGTTGGATGGAGAATGATGTGTGTATGGGTGAGGAAAATCGGAGGTTGC
>PZ670829_Type_sheep
ATTTTCAGTTTTTGGGGTGTGGGTATCGGAATGTGTGGTAGGTGATGTGTGTGTGTATGGGGGATGCCGAGGGGACCAGCGGTGCGGTGGTGTGTGTAGGCGTGAGAGTGTATCTGCAAGGGTGAGGGA
TGTGGGTGCAGCGAGTTAGAGGTGGTTCCATGTGGAATAGTGGGATTGGTACGTGATGGTTGGATGGGGGAATGATGTGTGTATGGGTGAGGAAAATCGGAGGTTGC
>PZ670830_CM7_sheep
ATTTTCAGTTTTTAGGGTGTGAGTATCGGAATGTATAGTAGGTGATGTATGTGTGTATGGGTGATGCCGAGGGGACCCGTGGTGCGGTGGTGTGTGTAGGCGTGAGAGTGTATCTGTAAGTGTGAGGGA
TGTAGCTGCAGTGAGTTAGAGATGGTTCCATGAGGAATAGTGGGATTGGTACACGATGGGTTGGATGGAGAATGATGTGTGTATGGGTGAGGAAAATCGGAGGTTGC
>PZ670831_CHG3_sheep
ATTTTCAGTTTTTAGGGTGTGAGTATCGGAATGTATAGTAGGTGATGTATGTGTGTATGGGGGATGCCGAGGGGACCCGCGGTGCGGTGGTGTGTGTAGGCGTGAGAGCGTATCTGTAAGTGTGAGGGA
TGTAGCTGCAGTGAGTTAGAGATGGTTCCATGAGGAATAGTGGGATTGGTACACGATGGGTTGGATGGAGAATGATGTGTGTATGGGTGAGGAAAATCGGAGGTTGC
>PZ670832_CHG1_sheep
ATTTTCAGTTTTTAGGGTGTGAGTATCGGAATGTACAGTAGGTGATGTATGTGTGTATGGGGGATGCCGAGGGGACCCGCGGTGCGGTGGTGTGTGTAGGCGTGAGAGTGTATCTGTAAGTGTGAGGGA
TGTAGCTGCAGTGAGTTAGAGATGGTTCCATGAGGAATAGTGGGATTGGTACACGATGGGTTGTATGGAGAATGATGTGTGTATGGGTGAGGAAAATCGGAGGTTGC
>PZ670833_CYG2_sheep
ATTTTCAGTTTTTAGGGTGTGAGTATCGGAATGTACAGTAGGTGATGTATGTGTGTATGGGTGATGCCGAGGGGACCCGCGGTGCGGTGGTGTGTGTAGGCGTGAGAGTGTATCTGTAAGTGTGAGGGA
TGTAGCTGCAGTGAGTTAGAGATGGTTCCATGAGGAATAGTGGGATTGGTACACGATGGGTTGGATGGAGAATGATGTGTGTATGGGTGAGGAAAATCGGAGGTTGC
>PZ670834_CHG5_goat
ATTTTCAGTTTTTAGGGTGTGAGTATCGGAATGTATAGTAGGTGATGTATGTGTGTATGGGGGATGCCGAGGGGACCCGCGGTGCGGTGGTGTGTGTAGGCGTGAGAGTGTATCTGTAAGTGTGAGGGA
TGTAGCTGCAGTGAGTTAGAGATGGTTCCATGAGGAATAGTGGGATTGGTACACGATGGGTTGTATGGAGAATGATGTGTGTATGGGTGAGGAAAATCGGAGGTTGC
>PZ670835_Type_goat
ATTTTCAGTTTTTGGGGTGTGGGTATCGGAATGTGTGGTAGGTGATGTGTGTGTGTATGGGGGATGCCGAGGGGACCAGCGGTGCGGTGGTGTGTGTAGGCGTGAGAGTGTATCTGCAAGGGTGAGGGA
TGTGGGTGCAGCGAGTTAGAGGTGGTTCCATGTGGAATAGTGGGATTGGTACGTGATGGTTGGATGGGGGAATGATGTGTGTATGGGTGAGGAAAATCGGAGGTTGC
>PZ670836_BEB6_goat
ATTTTCAGTTTTTAGGGTGTGAGTATCGGAATGTATAGTAGGTGATGTATGTGTGTATGGGTGATGCCGAGGGGACCCGCGGTGCGGTGGTGTGTGTAGGCGTGAGAGTGTATCTGTAAGTGTGAGGGA
TGTAGCTGCAGTGAGTTAGAGATGGTTCCATGAGGAATAGTGGGATTGGTACACGATGGGTTGGATGGAGAATGATGTGTGTATGGGTGAGGAAAATCGGAGGTTGC
>PZ670837_CHG1_goat
ATTTTCAGTTTTTAGGGTGTGAGTATCGGAATGTACAGTAGGTGATGTATGTGTGTATGGGGGATGCCGAGGGGACCCGCGGTGCGGTGGTGTGTGTAGGCGTGAGAGTGTATCTGTAAGTGTGAGGGA
TGTAGCTGCAGTGAGTTAGAGATGGTTCCATGAGGAATAGTGGGATTGGTACACGATGGGTTGTATGGAGAATGATGTGTGTATGGGTGAGGAAAATCGGAGGTTGC
>PZ846585_I_Cattle_Romania
ATTTTCAGTTTTTGGGGTGTGGGTATCGGAATGTGTGATAGGTGATGTGTGTGTGTATGGGGGATGCCGAGGGGACCAGCGGTGTGGTGGTGTGTGTAGGCGTGAGAGTGTATCTGTAAGGGTGAGGGA
TGTAGCTGCAGTGAGTTAGAGATGGTTCCATGAGGAATAGTGGGATTGGTACGTGATGGTTGGATGGGGGAATGATGTGTGTATGGGTGAGGAAAATCGGAGGTTGC
>PZ846586_BEB17_Cattle_Romania
---
TGCACTTTTTGGGGTGTGAGTATCGGAATGTGTAGTAGGTGATGTGTGTGTGTATGGGGGATGCCGAGGGGACCCGCGGTGCGGTGGTGTGTGTAGGCGTGAGAGCGTATCTGTAAGGGTGAGGGATGT
AGCTGCAGTGAGTTAGAGATGGTTCCATGAGGAATAGTGGGATTGGTACGTGATGGTTGGATGGGGGAATGATGTGTGTATGGGTGAGGAAAATCGGAGGTTGC
>PZ846587_J_Cattle_Romania
ATTTTCAGTTTTTGGGGTGTGGGTATCGGAATGTGTGG-
AGGTGATGTGTGTGTGTATGGGGGATGCCGAGGGGACCAGCGGTGTGGTGGTGTGTGTAGGCGTGAGAGTGTATCTGTAAGGGTGAGGGATGTAGCTGCAGTGAGTTAGAGATGGTTCCATGAGGAATA
GTGGGATTGGTACGTGATGGTTGGATGGGGGAATGATGTGTGTATGGGTGACGAAAATCGAAGGTTGC
>PZ860264_BEB8_Cattle_Romania
---
TTCAGTTTTTAGGGTGTGGGTATCGGAATGTATAGTAGGTGATGTATGTGTGTATGGGGGATGCCGAGGGGACCCGCGGTGCGGTGGTGTGTGTAGGCGTGAGAGCGTATCTGTAAGTGTGAGGGATGT
AGCTGCAGTGAGTTAGAGATGGTTCCATGAGGAATAGTGGGATTGGTACGTGATGGTTGGATGGGGGAATGATGTGTGTATGGGTGAGGAAAATCGGAGGTTGC
>PZ569993_BEB4_Cattle_Romania
A-----------TAAGGTG------------------------
AATGTGTGTGTGAATGGGGGATGCCGAGGGGACCAGCGGTGTGGTGGTGTGTGTAGGCGTGAGAGTGTATCTGTAAGGGTGAGGGATGTAGCTGCAGTGAGTTAGAGATGGTTCCATGAGGAATAGTGG
GATTGGTACGTGATGGTTGGATGGGGGAATGATGTGTGTATGGGTGAGGAAAATCGGAGGTTGC
```

Supplementary Data S2. Newick-formatted phylogenetic tree from the restricted ITS dataset (56 sequences; Groups 1, 2, 3, 8, and 14)

Machine-readable Newick representation of the Neighbor-Joining tree inferred from the restricted ITS dataset. Branch lengths represent evolutionary distances calculated using the Maximum Composite Likelihood method. Internal-node annotations retain the bootstrap-support values and MEGA12-exported standard-deviation fields obtained from 1,000 bootstrap replicates. Rate variation among sites was modeled using a gamma distribution.

(((((((((((((((((EU153585_BEB7_Cattle_USA:0.00435170485710662,KF543867_CM5_rhesus_macaque:0.00435170485710662)0.3130[stddev=1.46253205]:0.00
0337335601710288,KJ556988_HLJDV_Deer_China:0.00395457786470198)0.2170[stddev=1.28802174]:0.000511680953538756,PZ670833_CYG2_sheep:0.00376320
184423512)0.0800[stddev=0.85790442]:0.000554449413522508,PZ670836_BEB6_goat:-
0.000584949694114415)0.0830[stddev=0.85790442]:0.000298568073037547,PZ670828_BEB6_sheep:-
0.000298568073037547)0.0270[stddev=0.44271887]:0.000152460292614904,PX617835_Cattle_Algeria:-
0.000152460292614904)0.1170[stddev=0.98944429]:0.0000969282900211892,(PX617836_Cattle_Algeria:0.0,PZ670830_CM7_sheep:0.0)0.6260[stddev=1.534
92671]:0.00416552115067037)0.5230[stddev=1.57987341]:0.00528291954539549,PZ670831_CHG3_sheep:0.00340573838362064)0.4890[stddev=1.57987341]:0
.00208749257838587,(PZ670834_CHG5_goat:-
0.000521720002983344,(PZ670832_CHG1_sheep:0.0,PZ670837_CHG1_goat:0.0)0.5220[stddev=1.57987341]:0.00477801407016225)0.4680[stddev=1.57607106]
:0.00227884311040514)0.9970[stddev=0.31464265]:0.0343568634186808,KP262363_CHG10_Goat_China:0.0107100193855745)0.6620[stddev=1.49799866]:0.0
187161036329729,PZ860264_BEB8_Cattle_Romania:0.00886523670261154)0.3650[stddev=1.51789328]:0.00472192189576276,AY331007_BEB3_Cattle_USA:0.00
687401901745133)0.1830[stddev=1.21490740]:0.00266648997259458,(KT984495_BEB17_Cattle_Brazil:0.00370708978232955,PZ846586_BEB17_Cattle_Romani
a:0.0097030753192253)0.5650[stddev=1.56971335]:0.00514546186560551)0.4630[stddev=1.57607106]:0.0111032909982572,(AY331008_BEB4_Cattle_USA:8.
10181969228167E-
6,HM992510_CHN2_Human_China:0.00428583542399795)0.5090[stddev=1.58113883]:0.00120946954707271)0.1620[stddev=1.15931014]:0.00281493866644822,
PZ569993_BEB4_Cattle_Romania:0.0142944832467115)0.0950[stddev=0.90498619]:0.00181347331693013,JQ044399_BEB9_Cattle_USA:0.00168118617049248)0
.1530[stddev=1.12915898]:0.00181033914603022,((((PX617833_Cattle_Algeria:0.0,PZ846585_I_Cattle_Romania:0.0)0.6680[stddev=1.49799866]:0.0,MH7
32748_l_cattle:0.0)0.6670[stddev=1.49799866]:0.0,AF135836_I_Cattle_Germany:0.0)0.6660[stddev=1.49799866]:0.00362493773064447,(MN523335_SHR1_
Rat_China:0.00415142796584716,(AF135837_J_Cattle_Germany:-0.000049405912193418,(KX383638_JLD-VIII_Sika_deer_China:-
0.000049405912193418,(PX617832_Cattle_Algeria:-
0.0000658745495912249,PZ846587_J_Cattle_Romania:0.00874700597746124)0.4140[stddev=1.55531347]:0.000049405912193418)0.1780[stddev=1.18785521]
:0.000049405912193418)0.1210[stddev=1.02761861]:0.000910289261932681)0.2540[stddev=1.36930639]:0.000589895982834102)0.3560[stddev=1.5083103
1]:0.00097608986394631,(((((KJ867482_DeerEb3_white_tailed_deer_USA:0.00451955984475232,KJ867485_DeerEb6_white_tailed_deer_USA:-
0.000226725691549687)0.9540[stddev=0.68920244]:0.0087408348164758,KJ867484_DeerEb5_white_tailed_deer_USA:-
0.000034734829420555)0.6160[stddev=1.54240073]:0.0084287251385163,(KJ867487_DeerEb8_white_tailed_deer_USA:0.00916324379976166,KJ867488_DeerE
b9_white_tailed_deer_USA:0.00385969073994796)0.4410[stddev=1.56971335]:0.00279747026408507)0.9230[stddev=0.85790442]:0.0165124838879135,(MN5
23336_156_Rat:0.000343993333897192,(MK348513_MJ14_Binturong_China:-
0.000403485163597826,MK909573_SCR06_Rabbit_China:0.00469742240728806)0.5910[stddev=1.55531347]:0.00396327591534263)0.6290[stddev=1.53492671]
:0.0052210317977653 8)0.5330[stddev=1.57829021]:0.00618579235305846,(((((AY237212_WL4_Muskrat_US:0.00110495583071838,KF591686_12977_Deer_mous
e:0.00318787832248425)0.5850[stddev=1.56076904]:0.00284340561059347,(AY237214_5540_Muskrat:-
0.00110643239273678,DQ885584_PtEb_VIII_Cattle_Portugal:0.00974777927863596)0.9410[stddev=0.75099933]:0.00598785222912034)0.9400[stddev=0.750
99933]:0.0198640802611136,KF591684_13484_Woodchuck:0.00800613887605947)0.6550[stddev=1.50831031]:0.00604417297423507,KF591685_VL23_Deer_mous
e_USA:0.0105957456707643)0.8550[stddev=1.12915898]:0.0189229478781118,(AF101197_A_Human:0.000457564577582469,((FJ439681_S5_Human_Malawi:-
0.000775710163445219,PX617837_Cattle_Algeria:0.0137355895318566)0.6180[stddev=1.54240073]:0.00344465951750792,(PX617831_Cattle_Algeria:-
0.000011554389350481,(PZ670829_Type_sheep:-0.0000222199795201558,(PZ670835_Type_goat:-
0.0000427940346313989,(PV715457_Peru8_macaque:0.000179109748310384,(AF101200_D_Human:-0.000124980467568977,(MK809249_D_peacock:-
0.000241897679165765,(LC436503_WL2_deer_Korea:0.00476261399707392,PX617834_Cattle_Algeria:-
0.000468676753383683)0.5530[stddev=1.57321327]:0.000241897679165765)0.6400[stddev=1.51789328]:0.000124980467568977)0.6470[stddev=1.51789328]
:0.00412926939873394)0.6510[stddev=1.50831031]:0.00434187587218938)0.3210[stddev=1.47512711]:0.0000222199795201558)0.3210[stddev=1.47512711]
:0.000011554389350481)0.4940[stddev=1.58082257]:0.000933343132290015)0.6220[stddev=1.53492671]:0.00384697769063213)0.8100[stddev=1.24056439]
:0.015614838757137)0.2890[stddev=1.41985915]:0.00281406622173892)0.3610[stddev=0.00]:0.00900705329733086);

Figure S1. Restricted phylogenetic analysis of Groups 1, 2, 3, 8, and 14

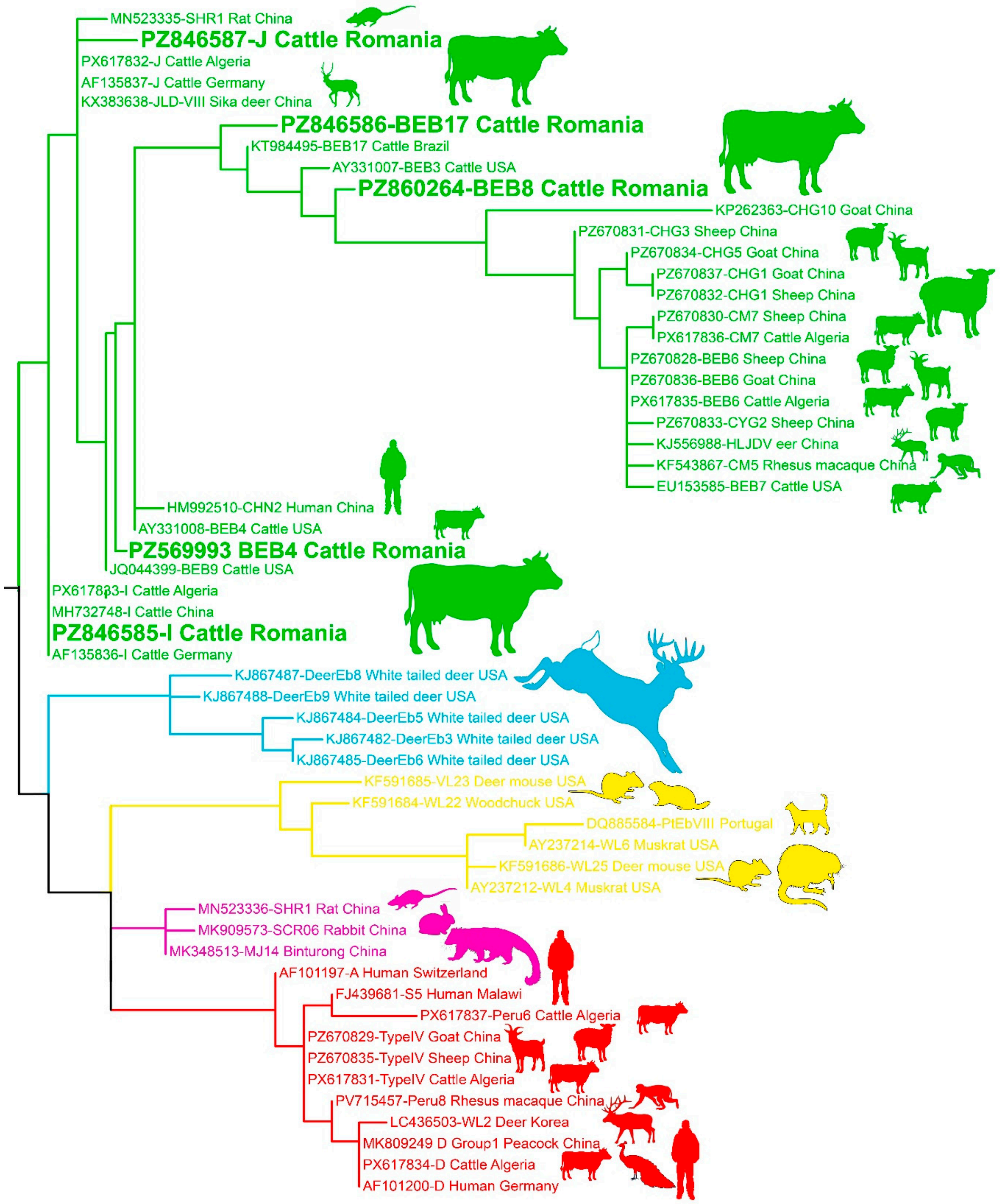

Figure S1. Phylogenetic relationships among 56 *E. bieneusi* ITS sequences representing Groups 1, 2, 3, 8, and 14. The Neighbor-Joining tree was inferred using Maximum Composite Likelihood distances, with rate variation among sites modeled using a gamma distribution; node support was assessed using 1,000 bootstrap replicates in MEGA12. Group 2 is shown in green, Group 1 in red, Group 3 in yellow, Group 8 in blue/cyan, and Group 14 in magenta. Romanian cattle-derived sequences are highlighted in bold green type, and host silhouettes indicate the host origin of representative sequences.
